# What Narcissists Look Like and Why It’s Important

**DOI:** 10.1177/01461672251339014

**Published:** 2025-05-22

**Authors:** Sarah Smith, Travis Proulx, Geoffrey Haddock

**Affiliations:** 1 Cardiff University, UK

**Keywords:** narcissism, mental representations, lay perceptions, interpersonal judgments

## Abstract

Prior research investigating public perceptions of narcissistic individuals has relied on participant ratings of researcher-selected dimensions or character vignettes, limiting generalizability and ecological validity. Using reverse correlation—a bottom-up, participant-driven method—we examined how people visually represent narcissists, and the consequences of these representations on attributional perceptions (e.g., trust, leadership, attraction). As narcissism is commonly perceived in terms of selfishness or vanity, participants generated facial images where the selfish (Experiment 1) or vain (Experiment 2) dimensions of narcissism were made salient—resulting in selfish-narcissistic versus non-selfish faces and vain-narcissistic versus non-vain faces. Experiment 3 directly compared representations of selfish- and vain-narcissistic faces and their non-narcissistic counterparts. While narcissistic facial images were generally perceived unfavorably by naïve raters, the vain-narcissistic face was seen as more agentic (e.g., competent) and attractive than the selfish-narcissistic face. Narcissistic (vs. non-narcissistic) raters also viewed the vain-narcissistic face more favorably, an effect mediated by perceived similarity.

Judgments based on facial appearances are deeply ingrained. Often occurring within milliseconds (Willis & Todorov, 2006), face-based judgments are linked with increased activity in the amygdala, a brain region linked with impression formation (Rule et al., 2011). Indeed, the morphological properties in a human face can reliably signal personality and behavioral tendencies (Kachur et al., 2020). Recognizing the functional consequences of facially signaled attributional perceptions, researchers have examined visual representations of categories such as atheists (Brown-Iannuzzi et al., 2018) and Liberals/Conservatives (Proulx et al., 2023). Yet, to our knowledge, research has not assessed lay perceptions of narcissism at the facial level, or, simply put, what people think narcissists look like. This is despite the public’s magnetism toward narcissism and the proliferation of popular discourse regarding narcissism. For instance, a recent book (Durvasala, 2024) billed as a “survival guide” for protecting and healing oneself from the daily harms of narcissism became a New York Times bestseller. Further, social media is brimming with content about narcissism. On TikTok, the hashtag #narcissist had over 12 billion views as of December 2023.

Understanding how people mentally represent narcissists is important for broadening social attributions associated with narcissism and their implications. Yet, limited research has explored how people mentally represent narcissists and the outcomes associated with these representations. Across three preregistered experiments, we utilized reverse correlation (Dotsch & Todorov, 2012) to generate lay representations of narcissistic and non-narcissistic faces and examined subsequent judgments of these faces on meaningful attributes (e.g., warmth, competence, personal values) and social outcomes (e.g., perceived leadership, trustworthiness, attraction).

## Conceptualizations of Narcissism

Although academic conceptualizations of narcissism have often been heavily contested, it is now understood as a construct grounded by a core dimension termed antagonism, rivalry, or entitlement (Miller et al., 2021). This *selfish core* of narcissism (Campbell, 2022), is thought to represent the binding factor shared by narcissistic expressions. For example, trifactor models of narcissism (Crowe et al., 2019; Krizan & Herlache, 2018) posit that this selfish core is common to both grandiose and vulnerable narcissism, with the former characterized by arrogance and high self-esteem, and the latter by distrust and low self-esteem. This distinction has generated considerable attention, with research demonstrating that grandiose narcissists are particularly likely to self-enhance and self-promote, with behavior motivated by an approach-focused orientation. In contrast, vulnerable narcissists are more likely to endorse interpersonal hostility and defensive behavior, with actions motivated by an avoidance-focused orientation (see Miller et al., 2021; Sedikides, 2021).

Research is mixed regarding whether laypersons perceive narcissists as more antagonistic (e.g., selfish), grandiose (e.g., vain), or vulnerable (e.g., insecure). Some studies suggest that participants view grandiosity as the defining characteristic of narcissism (Carlson et al., 2012; Miller et al., 2018), while other research highlights beliefs in antagonism and defensiveness (e.g., Park & Colvin, 2014; Stanton et al., 2018). More recently, Smith et al. (2024) asked lay participants to freely describe their definition of narcissism. A thematic analysis of these definitions revealed that, while some respondents referenced insecurity and emotional fragility—aligning their conceptualizations with vulnerable narcissism, the most frequently referenced themes associated with narcissism were *selfishness* and *vanity*, consistent with previous investigations of dominant lay perceptions of narcissism (e.g., Miller et al., 2018).

However, missing from this work is an understanding of how people visualize narcissists. In extant research, participants rated either hypothetical narcissistic characters or real-world narcissistic acquaintances. This approach may lead participants to ascribe more negative attributes simply due to exposure to the pejorative term “narcissist.” One way to counter this methodological limitation is via the use of reverse correlation—a method for generating facial images of a social group member—as the term “narcissist” and any associated features are completely omitted from the rating process.

## Visual Representations of Narcissists

To date, the limited research examining visualizations of narcissism has focused on how people detect narcissism in faces. These studies have examined how narcissism is manifested in facial areas (e.g., eyebrows; Giacomin & Rule, 2019) and participants’ ability to detect narcissism in composite facial images (Alper et al., 2021; Holtzman, 2011). These latter studies rely on facial composites, created by morphing faces of individuals extremely high or low in narcissism. While informative, this approach has been criticized for its lack of methodological transparency and external validity (Bovet et al., 2022). Furthermore, the faces selected represent visual representations of *researcher-selected* indices of narcissists, rather than *participant-generated* representations of the public image of narcissists.

Reverse correlation, on the other hand, represents a bottom-up, participant-driven method that offers an unconstrained visualization of facial information prototypical of social categories (Brinkman et al., 2017). This method comprises two stages: first, one sample of participants generates a facial image they perceive as representative of a group member (e.g., a narcissist). The individually generated images are then averaged across participants, creating one classification facial image emblematic of a prototypical category member. Second, another sample of participants, *unaware* of how the prototypical face was generated, evaluates the image (usually alongside its opposite, e.g., a non-narcissist) on outcome measures.

Utilizing reverse correlation to visualize representations of narcissism has important advantages. First, it allows for the unbiased generation of facial characteristics that drive meaningful social outcomes. Second, as the faces generated by one sample are *verified* as a category member by a naïve sample, it offers a more generalizable and ecologically valid method relative to facial composite procedures.

## The Present Research

The focus of our research was to examine lay perceptions of narcissism as represented facially and the consequences of these representations on attributional evaluations. Put differently, we assessed what people think narcissistic individuals (and non-narcissistic individuals) *look like* and whether people differentiate between visual representations of narcissists and non-narcissists. As narcissists tend to be conceptualized as primarily antagonistic or grandiose, we assessed visual representations of narcissists in two ways, where either the selfish (Experiment 1) or vain (Experiment 2) facets were made salient. The focus on selfishness and vanity aligns with research highlighting the importance of these dimensions in how laypeople define narcissism (Smith et al., 2024). As such, Experiment 1 considers representations of what we refer to as the “*selfish-narcissistic*” and the “*non-selfish*” faces. Similarly, Experiment 2 considers representations of what we refer to as the “*vain-narcissistic*” and the “*non-vain*” faces. We were interested in assessing whether focusing on selfishness or vanity would lead to different visual representations, with unique evaluative consequences.

Experiment 1 compared evaluations of the selfish-narcissist and non-selfish images on personality attributes, values, morality, and their suitability for various professions, whereas Experiment 2 compared evaluations of the vain-narcissist and non-vain images on the same outcomes. Experiment 3 examined perceptions of both the selfish- and vain-narcissist faces, and their non-narcissistic counterparts, on dimensions related to physical/romantic attraction. Furthermore, as narcissists tend to hold more favorable views of other narcissists (*narcissistic tolerance*, Hart & Adams, 2014), across all experiments we conducted exploratory analyses examining whether rater narcissism was positively associated with more favorable evaluations of the narcissistic faces.

We preregistered all experiments on the OSF (Experiment 1: [https://osf.io/dqmy9], Experiment 2: [https://osf.io/j5s26], Experiment 3: [https://osf.io/cs9hy]). All data, analysis code, and research materials are available at [https://osf.io/4t5az/files/osfstorage]. Data were analyzed using R, version 4.2.3 (R Core Team, 2023) and jamovi version 2.3 (The Jamovi Project, 2023).

## Experiment 1—What Do People Think a Selfish-narcissist Looks Like?

Experiment 1 examined how people visually represent narcissistic (vs. non-narcissistic) individuals when narcissism’s *selfishness* component is salient. One sample of participants (generators) completed a task that resulted in selfish-narcissist and non-selfish classification images. Next, another independent sample evaluated both images. We examined whether people hold different mental images of selfish-narcissistic and non-selfish individuals, and whether a new sample of naïve participants would rate these images differently on a number of attributes (including perceived narcissism, selfishness, vanity, kindness, masculinity, age, political orientation, self-esteem), Big Five traits, interpersonal qualities (warmth, competence, liking and success), personal values, moral behaviors, and workplace roles.

We expected the selfish-narcissistic face to be judged less favorably than the non-selfish face. Based on prior research regarding lay perceptions of narcissistic acquaintances (Smith et al., 2024), we expected participants to perceive the selfish-narcissistic face as placing more importance on self-enhancement values (e.g., wealth, power) and less on self-transcendence (e.g., honesty, equality), openness (e.g., freedom, curiosity), and conservation (e.g., politeness, obedience) values, be politically conservative, and as less moral, relative to the non-selfish face.

We also considered consequences regarding how people would interact with the selfish-narcissistic (vs. non-selfish) images. Specifically, participants indicated their likelihood of voting for each of the images to lead their country, how much they would trust each image to look after a loved one, and how comfortable they would feel if trapped in an elevator with each individual. We expected the selfish-narcissistic face to be ascribed lower ratings across these items.

Finally, participants reported how much they shared in common with each image. Faces that resemble a rater’s own face are evaluated more positively relative to non-self-resembling faces (Bailenson et al., 2008). We expected the selfish-narcissistic (vs. non-selfish) face to be ascribed lower levels of perceived similarity, but that higher (vs. lower) rater self-reported narcissism would predict greater levels of perceived similarity with the selfish-narcissistic face. This prediction was informed by research linking similarity perception to increased tolerance of other narcissists (Burton et al., 2017).

### Method

#### Image Generation Phase

##### Participants

We recruited 155 Cardiff University students. Twenty-eight participants were excluded for failing attention check trials during the reverse correlation task and/or failing an attention check item during the survey (see Materials and Procedure). This resulted in a final sample of 127 (109 females, 17 males, 1 prefer not to say; *M*_age_ = 19.98, *SD*_age_ = 2.05).

##### Materials and Procedure

Participants completed the reverse correlation task using PsychoPy (Peirce et al., 2019). First, participants were assigned randomly to generate the face of either a narcissistic or selfless individual. The generated selfless face was not intended to be utilized in the subsequent image rating phase, but was used in a separate line of work to confirm that the faces *not selected* as narcissistic adequately approximated the opposite of that social category (see Image Processing).

**Figure 1.**
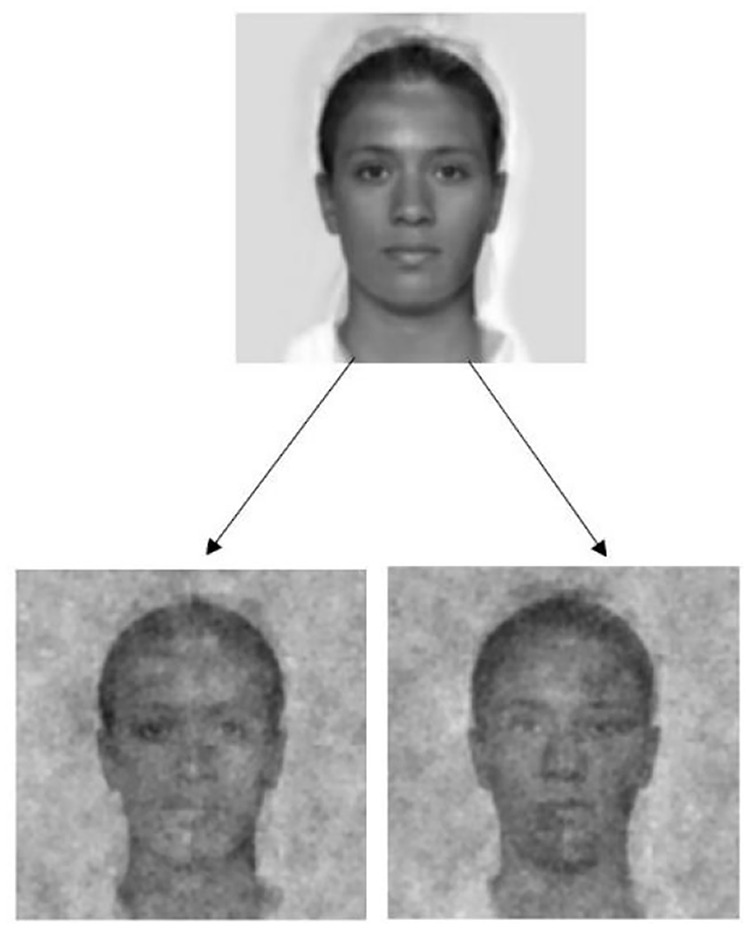
The base image used in the reverse correlation task and example of an image pair.

Before the task, participants in the narcissistic condition (*N* = 65) were instructed that “narcissism is a trait which reflects egocentric exceptionalism and social selfishness, that is, superiority and entitlement beliefs accompanied by indifference or apathy toward others” (Sedikides, 2021, p. 68). The task consisted of 400 trials; participants could take a break after every 100 trials. For each trial, participants were presented with two images and asked to select the image that “best represents a narcissist to you.” One image was a base face superimposed with a random white noise pattern, the second image displayed the reverse noise pattern superimposed onto the same base face (see Figure 1). The random noise patterns were added using the R rcicr package (v0.3.4.1; Dotsch, 2015). The base face (a morphed composite of a Black female, a Black male, a White female, and a White male) was generated using images from the Face Research Lab (DeBruine & Jones, 2017). As suggested by Brinkman et al. (2017), a Gaussian blur was used to smooth the base face image for it to best match the power spectrum of the added noise.

Ten attention checks were interspersed within the task. For each check, participants were shown the faces of an adult and a child and asked to select the child’s face. Participants had to pass at least 50% of the attention checks for their data to remain in subsequent analyses. This threshold has been used in other reverse correlation research (Han et al., 2023).

Following the reverse correlation task, participants were redirected to Qualtrics to complete four narcissism scales presented in random order. These included the Narcissistic Personality Inventory (NPI-13; Gentile et al., 2013), the Vulnerable Narcissism facet of the Five-Factor Narcissism Inventory (FFNI; Glover et al., 2012), the Communal Narcissism Inventory (Gebauer et al., 2012), and the Narcissistic Admiration and Rivalry Questionnaire (NARQ-S; (Leckelt et al., 2018). The NPI-13 included an attention check item that required participants to select a certain number on a scale. Other than the NPI-13, the inclusion of these scales was not relevant to the subsequent analyses reported here, but rather for exploratory purposes, to compare classification images generated by participants with high (vs. low) scores (see Figures S1–S4).

Next, participants completed explicit (Robins et al., 2001) and implicit measures of self-esteem (Gebauer et al., 2008), and a shortened version of Schwartz’s Value Survey (SVS; Schwartz, 1992). We did not analyze these data in the context of the current paper. Lastly, participants completed demographic questions.

#### Image Processing

**Figure 2.**
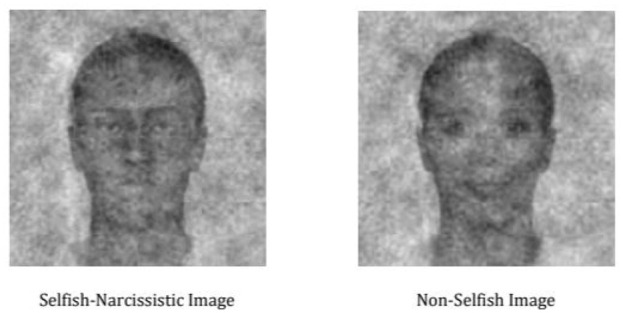
Average classification images (selfishness definition).

Using the R rcicr package (v0.3.4.1; Dotsch, 2015), we computed the average narcissistic classification image (i.e., the selfish-narcissistic face) by superimposing the averaged noise patterns selected by individual participants across trials onto the base face image. The non-narcissistic classification image (i.e., the non-selfish face) was created using the same process, with one exception: we averaged the noise patterns across images that were *not* selected by individual participants. The resulting images are displayed in Figure 2. This processing method is common (e.g., Brown-Iannuzzi et al., 2018), and evidence suggests that classification images generated using non-selected images represent robust portrayals of the opposite of the given category (Dotsch & Todorov, 2012; Lick et al., 2013). Nonetheless, to ensure that the faces not selected as narcissistic sufficiently approximated a selfless face, a separate pilot study (Figure S5 and Table S1) found that the selfless and non-selfish faces elicited identical ratings across all dimensions of interest.

#### Image Rating Phase

##### Participants

We recruited Cardiff University students (*n* = 114) and UK residents via Prolific (*n* = 90). Seven participants were excluded for failing an attention check item, resulting in final sample of 197 (125 females, 64 males, 4 others, 4 prefer not to say; *M*_age_ = 26.92, *SD*_age_ = 12.18).

We conducted an a priori power analysis (G*Power 3.1; Faul et al., 2009) to determine the sample size needed to achieve enough power (0.80) to detect small effect sizes (*r* = .20) between participant narcissism and perceptions of the faces at *p* < .05 (two tailed). Results indicated that a sample of 193 was sufficient.

##### Materials and Procedure

Participants completed the task via Qualtrics. After providing consent, participants were informed that they would make judgments about faces. Participants evaluated each face individually; no information was provided about the faces or how they were created. The session included different phases. In all phases, questions were presented in a random order, each on a separate screen. For the first two phases, participants rated both the selfish-narcissistic and non-selfish faces, and a filler face. The filler face was a neutral noise-altered base face image included so that the comparison of the two critical images would not be salient (Brown-Iannuzzi et al., 2018).

First, participants rated the faces on narcissism (1 = *not at all*, 7 = *extremely*), political orientation (1 = *extremely liberal*, 7 = *extremely conservative*) and age. Second, participants rated the faces on selfishness, vanity, kindness, the Big Five traits, interpersonal traits (warmth, competence, liking, and success; 1 = *not at all*, 7 = *extremely*), and self-esteem (1 = *extremely low*, 7 = *extremely high*).

Third, participants rated how important they perceived four different value types to be for both faces. We used a shortened version of the SVS (Schwartz, 1992), where participants responded for each value type using a sliding scale (0 = *less important to them than to the average person living in the UK*; 100 = *more important to them than to the average person living in the UK*).

Fourth, participants judged the likelihood of the selfish-narcissistic and non-selfish faces to have committed various moral and immoral acts (Brown-Iannuzzi et al., 2018). These acts included four moral (e.g., Left food out for a stray cat) and four immoral (e.g., Kicked a dog for no reason) behaviors. All items were presented in a random order (1 = *not at all likely*, 7 = e*xtremely likely*).

Fifth, participants judged the two images concerning workplace perceptions (see Han et al., 2023). Two judgments concerned career suitability, with participants indicating how well suited each image was for a career in (a) corporate management and (b) health services (1 = *not at all well suited*, 7 = *extremely well suited*). Two other judgments related to workplace relations; participants rated how desirable each image would be to work *with* and to work *for* (1 = *strongly disagree*, 7 = *strongly agree*).

Next, participants indicated their perceptions of the two images’ leadership qualities (i.e., the likelihood that they would vote for each image to lead their country’s government [1 = *not at all likely*, 7 = *extremely likely*]), trustworthiness (i.e., the extent that they would trust each image to look after a loved one needing care [1 = *not at all*, 7 = *a great deal*]), physical proximity comfort (i.e., how comfortable they would feel if trapped in an elevator with each image [1 = *not at all comfortable*, 7 = *extremely comfortable*]), and similarity (i.e., how much they shared in common [1 = *nothing at all*, 7 = *a great deal*]). In all phases, questions were presented in a random order and on a separate screen.

Finally, participants completed the NPI-13 (Gentile et al., 2013; *M* = 3.17, *SD* = 1.06, α = .87), NARQ-S (Leckelt et al., 2018; *M* = 2.77, *SD* = 1.20, α = .74), and demographic information.

### Results

We first report our preregistered testing for differences between the faces, where we used Bonferroni corrected paired *t*-tests. The results, along with descriptive scores and analyses of absolute differences, are presented in Table 1. Second, we report our preregistered testing for associations between rater narcissism and evaluations of the selfish-narcissistic face via a series of Bonferroni corrected Pearson’s correlations.

**Table 1.** Absolute Trait Ratings of the Selfish-narcissistic and Non-selfish Faces.

	Selfish-narcissist	Non-selfish	*t*-tests		
Outcome measure	*M* (*SD*)	*M* (*SD*)	*t*	Cohen’s *d*	*p*
Attributes					
Narcissistic	4.58** (1.57)	3.15** (1.55)	9.45	0.67	<.001
Selfish	4.93** (1.47)	2.98** (1.38)	13.74	0.98	<.001
Vain	3.83 (1.78)	3.43** (1.43)	2.51	0.18	.013
Masculine	5.57** (1.41)	2.39** (1.25)	22.23	1.58	<.001
Politics	4.21* (1.43)	3.43** (1.29)	5.95	0.42	<.001
Self-esteem	3.79* (1.43)	4.75** (1.17)	−6.96	−0.50	<.001
Kind	2.47** (1.22)	5.24** (1.31)	−19.75	−1.41	<.001
Warm	2.15** (1.22)	5.26** (1.33)	−21.71	−1.55	<.001
Likable	2.60** (1.27)	4.97** (1.50)	−15.85	−1.13	<.001
Competent	3.86 (1.35)	4.39** (1.29)	−3.83	−0.27	<.001
Successful	3.45** (1.33)	4.64** (1.20)	−9.70	−0.69	<.001
Open	2.60** (1.29)	5.15** (1.20)	−18.11	−1.29	<.001
Conscientious	3.37** (1.34)	4.49** (1.21)	−8.52	−0.61	<.001
Extraverted	2.89** (1.36)	5.09** (1.33)	−15.15	−1.08	<.001
Agreeable	2.46** (1.29)	5.13** (1.37)	−18.60	−1.33	<.001
Neurotic	4.05 (1.53)	3.76* (1.61)	1.73	0.12	.085
Age	25.44 (5.96)	27.47 (6.76)	−3.52	−0.25	<.001
Values					
Self-transcendence	32.64** (20.43)	64.34** (18.67)	−16.05	−1.14	<.001
Self-enhancement	61.03 ** (21.59)	46.66* (18.97)	6.50	0.46	<.001
Openness	39.68 ** (20.95)	63.55** (17.77)	−12.14	−0.87	<.001
Conservation	35.82 ** (22.97)	58.23** (21.31)	−9.41	−0.67	<.001
Morality	−1.01** (2.01)	2.68** (1.98)	−16.04	−1.14	<.001
Workplace					
Corporate	3.92 (1.81)	4.18 (1.43)	−1.42	−0.10	.157
Health	3.10 ** (1.54)	5.39** (1.39)	−15.20	−1.08	<.001
Boss	2.81** (1.56)	4.89** (1.61)	−11.82	−0.84	<.001
Colleague	3.09** (1.57)	5.07** (1.48)	−11.48	−0.82	<.001
Behavioral					
Prime Minister	2.78** (1.55)	4.23* (1.55)	−9.07	−0.65	<.001
Trust	2.95** (1.56)	5.15** (1.56)	−13.55	−0.97	<.001
Close proximity	3.07** (1.57)	5.04** (1.46)	−12.00	−0.86	<.001
Similarity	2.81** (1.31)	4.13 (1.39)	−9.22	−0.66	<.001

* *p* < .05, **p < .01 difference from scale midpoint.

#### Comparing the Selfish-narcissist and Non-selfish Faces

##### Attributes

As predicted, participants considered the selfish-narcissistic face as more narcissistic, selfish, and conservative, and less warm, kind, likable, competent, successful, open, agreeable, conscientious, extraverted, and lower in self-esteem (all *p*s < .001). The selfish-narcissistic face was also rated as more masculine and younger (*p* < .001). The largest effect size differences were observed for masculinity (*d* = 1.58, 95% CI [1.35, 1.81], warmth (*d* = −1.55, 95% CI [−1.78, −1.32]), and agreeableness (*d* = −1.33, 95% CI [−1.55, −1.11]), indicating especially pronounced contrasts on these attributes. We found no significant differences in ratings of vanity and neuroticism (*p*s > .013).

##### Values

A 2 (face type) × 4 (value type) repeated measures ANOVA revealed a main effect of face type, *F*(1, 196) = 164.43, *p* < .001, $\eta_{p}^{2}$ = .456. Higher value importance ratings were attributed to the non-selfish than selfish-narcissistic face. There was also a significant main effect of value type, *F*(2.68, 526.00) = 15.28, *p* < .001, $\eta_{p}^{2}$ = .072. Self-transcendence and conservation values were seen as less important than self-enhancement and openness values (all *p*s < .016).

Importantly, these effects were qualified by a significant interaction, *F*(2.46, 481.33) = 104.55, *p* < .001, $\eta_{p}^{2}$ = .348. Regarding the self-transcendence and self-enhancement dimension, the selfish-narcissistic (vs. non-selfish) face was perceived as valuing self-transcendence less (*p* < .001, $\eta_{p}^{2}$ = .568), and self-enhancement more (*p* < .001, $\eta_{p}^{2}$ = .178). Regarding the openness and conservation dimension, the selfish-narcissistic (vs. non-selfish) face was perceived as attaching less importance to openness (*p* < .001, $\eta_{p}^{2}$ = .429) and conservation values (*p* < .001, $\eta_{p}^{2}$ = .311).

##### Morality

We created an index of moral behavior for both faces by subtracting each face’s average immorality score from their average morality score. The selfish-narcissistic (vs. non-selfish) face was judged as less moral (*p* < .001, Cohen’s *d* = −1.14).

##### Workplace Roles

Regarding workplace suitability, the selfish-narcissistic (vs. non-selfish) face was judged as less suited for a career in health services (*p* < .001, Cohen’s *d* = −1.08). We found no effect on suitability for corporate management (*p* = .157). For workplace relations, the selfish-narcissistic (vs. non-selfish) face was seen as a less desirable work colleague (*p* < .001, Cohen’s *d* = −0.82) and boss (*p* < .001, Cohen’s *d* = −0.84).

##### Leadership, Trustworthiness, Comfort with Physical Closeness, and Similarity

Participants were less likely to vote for the selfish-narcissistic (vs. non-selfish) face to be Prime Minister (*p* < .001, Cohen’s *d* = −0.65) and to trust the selfish-narcissistic to look after a loved one (*p* < .001, Cohen’s *d* = −0.97). Additionally, participants reported feeling less comfortable if trapped in an elevator with the selfish-narcissistic (vs. non-selfish) face (*p* < .001, Cohen’s *d* = −0.86) and reported sharing less in common with the selfish-narcissistic face (*p* < .001, Cohen’s *d* = −0.66).

#### Associations Between Rater Narcissism and Evaluations of the Selfish-narcissist

Next, we assessed whether rater narcissism was linked with perceptions of the selfish-narcissistic face. Greater rater narcissism was positively associated with perceived narcissism (*r*(195) = .22, *p* = .002), neuroticism (*r*(195) = .26, *p* < .001), and vanity (*r*(195) = .28, *p* < .001). Rater narcissism was unrelated to perceptions of any other outcomes (all *p*s > .014). Therefore, in contrast with predictions, individuals higher in narcissism did not perceive themselves as sharing more in common with the selfish-narcissist. Accordingly, we did not conduct preregistered mediation analyses testing whether similarity mediates the relationship between rater narcissism and evaluations.

### Discussion

This experiment examined visual representations of selfish-narcissistic and non-selfish individuals, testing whether naïve raters would differentially evaluate these representations. Additionally, we assessed the relationship between raters’ narcissism and perceptions of the selfish-narcissistic face.

As expected, the selfish-narcissistic face was judged less favorably than the non-selfish face. The selfish-narcissistic face was seen as more narcissistic and selfish, and as less warm, likable, kind, agreeable, open, conscientious, and moral. We also hypothesized and found that the selfish-narcissistic face would be seen as more self-enhancing and less self-transcending in terms of their value orientation.

However, raters’ own narcissism was not correlated with more favorable evaluations of the selfish-narcissistic face. This could be linked with the reverse correlation method; perhaps mutual liking between narcissists does not apply when narcissism is communicated facially. Alternatively, this pattern might reflect the definition provided to our generators, which emphasized narcissists’ pejorative interpersonal qualities (i.e., selfishness), omitting the grandiose/admirative (i.e., vain) qualities that can be perceived more positively. This suggests that the inclusion of more favorable components may be important for establishing this link, potentially, as in Burton et al. (2017), via perceived similarity. Experiment 2 tested this possibility by generating new narcissistic and non-narcissistic faces using a definition of narcissism that accounts for the vain aspects of narcissism.

## Experiment 2—What Do People Think a Vain-narcissist Looks Like?

Experiment 2 utilized the same methodology as Experiment 1, with one fundamental difference: we provided generators with a definition of narcissism that emphasized the *vanity* component. The definition we used was directly adopted from the Single Item Narcissism Scale (SINS; Konrath et al., 2014), which instructs that “narcissism means being egotistical, self-focused, and vain”. Thus, while both Experiment 1 and 2’s definitions highlighted the superiority/egocentric aspects of narcissism, Experiment 2’s definition additionally emphasized narcissistic vanity. This is important because vanity is commonly reflected in models and lay definitions of narcissism (Miller et al., 2021; Smith et al., 2024).

Experiment 2 tested whether emphasizing the vanity component (a) influences visual representations of narcissism, (b) elicits distinct subsequent evaluations of vain-narcissistic and non-vain classification images, and (c) activates narcissistic tolerance among raters with greater self-reported narcissism. Our preregistered testing compared relative differences between the vain-narcissistic (vs. non-vain) faces generated by Experiment 2 generators, as well as relative differences between the *vain-*narcissistic and non-vain faces and the *selfish-*narcissistic and non-selfish faces (from Experiment 1). As an exploratory investigation, we examined the relationship between rater narcissism and evaluations of the vain-narcissistic face.

### Method

#### Image Generation Phase

##### Participants

We recruited 130 Cardiff University students. Twenty-three participants were excluded for failing the attention check criteria during the reverse correlation task and seven for failing an attention check item during the Qualtrics survey. This resulted in a final sample of 100 (80 females, 14 males, 6 others; *M*_age_ = 19.38, *SD*_age_ = 1.43).

##### Materials and Procedure

Other than the definition provided to generators, the Materials and Procedure were identical to Experiment 1. Participants were instructed that “narcissism means being egotistical, self-focused, and vain” (Konrath et al., 2014).

#### Image Processing

**Figure 3.**
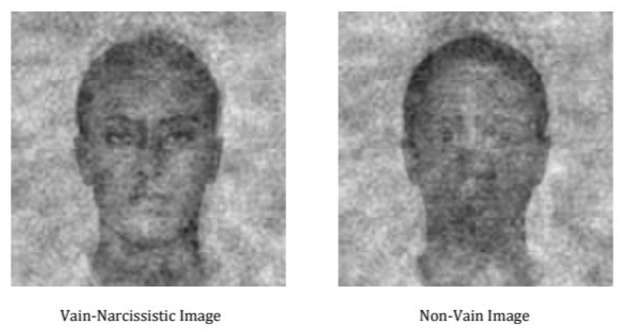
Average classification images (vanity definition).

The classification images were created in the same way as in Experiment 1. The resulting images are displayed in Figure 3.

#### Image Rating Phase

##### Participants

We recruited Cardiff University students (*n* = 135) and UK residents via Prolific (*n* = 85). Five participants were excluded for failing an attention check item, resulting in a final sample of 215 (152 females, 60 males, 3 others; *M*_age_ = 26.66, *SD*_age_ = 11.57).

We conducted an a priori power analysis (G*Power 3.1; Faul et al., 2009) using the “ANOVA: Repeated measures, within-between interaction” method. Results suggested that 138 participants were needed to ensure 80% statistical power for a small effect size (*f* = 0.10; *p* < .05). Regarding our exploratory analyses of correlations between rater narcissism and perceptions of the narcissistic face, G*Power determined that a sample of 193 was sufficient to achieve enough power (0.80) to detect small effect sizes (*r* = .20; *p* < .05; two tailed).

##### Materials and Procedure

The Materials and Procedure were identical across Experiments 1 and 2. Again, the participants completed the NPI-13 (*M* = 3.29, *SD* = 0.98, α = .87), NARQ (*M* = 2.76, *SD* = 1.02, α = .76), and demographic information.

### Results

First, we report our preregistered testing for relative differences between the faces via Bonferroni corrected paired *t*-tests. The results, along with descriptive scores and exploratory analyses of absolute differences on ratings, are presented in Table 2. Second, we report exploratory testing of the relationship between rater narcissism and evaluations of the vain-narcissistic face via Bonferroni corrected Pearson’s correlations and exploratory mediation analyses.

**Table 2.** Absolute Trait Ratings of the Vain-narcissistic and Non-vain Faces.

	Vain-narcissistic	Non-vain	*t*-tests		
Outcome measure	*M* (*SD*)	*M* (*SD*)	*t*	Cohen’s *d*	*p*
Attributes					
Narcissistic	5.33** (1.15)	2.84** (1.35)	20.78	1.42	<.001
Selfish	5.51** (1.20)	2.87** (1.38)	20.17	1.38	<.001
Vain	5.13** (1.48)	2.33** (1.18)	20.20	1.38	<.001
Masculine	4.48** (1.46)	4.02 (1.34)	3.43	0.23	<.001
Politics	4.51** (1.45)	3.39** (1.10)	8.65	0.59	<.001
Self-esteem	4.86** (1.50)	3.09** (1.24)	12.23	0.83	<.001
Kind	2.17** (1.22)	4.67** (1.29)	−19.51	−1.33	<.001
Warm	1.88** (1.14)	4.27* (1.38)	−18.90	−1.29	<.001
Likable	2.37** (1.02)	4.40** (1.35)	−17.17	−1.17	<.001
Competent	4.32** (1.44)	3.67** (1.26)	4.67	0.32	<.001
Successful	4.17 (1.38)	3.37** (1.24)	6.27	0.43	<.001
Open	2.80** (1.36)	3.70* (1.38)	−6.94	−0.47	<.001
Conscientious	3.36** (1.42)	4.22* (1.31)	−5.87	−0.40	<.001
Extraverted	3.76* (1.52)	2.93** (1.36)	6.10	0.42	<.001
Agreeable	2.19** (1.10)	4.53** (1.34)	−18.66	−1.27	<.001
Neurotic	4.37** (1.58)	3.87 (1.47)	3.20	0.22	.002
Age	26.92 (5.36)	25.42 (9.94)	2.33	0.16	.021
Values					
Self-transcendence	28.30** (18.78)	56.56** (20.35)	−14.99	−1.02	<.001
Self-enhancement	69.97** (22.45)	37.69** (18.83)	15.10	1.03	<.001
Openness	41.93** (22.74)	48.10 (21.90)	−3.09	−0.21	.002
Conservation	38.62** (26.72)	55.07* (23.26)	−6.56	−0.45	<.001
Morality	−1.57** (1.74)	2.43** (1.82)	−20.82	−1.42	<.001
Workplace					
Corporate	4.66** (1.72)	3.38** (1.40)	7.70	0.53	<.001
Health	2.90** (1.47)	4.61** (1.43)	−12.49	−0.85	<.001
Boss	2.53** (1.49)	4.01 (1.52)	−10.35	−0.71	<.001
Colleague	2.68** (1.45)	4.60** (1.42)	−13.46	−0.92	<.001
Behavioral					
Prime Minister	2.60** (1.55)	3.31** (1.54)	−5.05	−0.35	<.001
Trust	2.77** (1.44)	4.51** (1.46)	−13.51	−0.92	<.001
Close proximity	2.78** (1.45)	4.57** (1.45)	−13.92	−0.95	<.001
Similarity	2.72** (1.27)	3.52** (1.33)	−7.12	−0.49	<.001

* *p* < .05, **p < .01 difference from scale midpoint.

Next, we present our preregistered testing of relative differences between the narcissistic and non-narcissistic faces generated using the selfish and vain definitions. We tested these via 2 (face type: narcissistic, non-narcissistic) × 2 (definition: selfish, vain) mixed ANOVAs. We follow up any significant interactions by conducting pairwise comparisons between the two narcissistic faces and the two non-narcissistic faces.

#### Comparing the Vain-narcissistic and Non-vain Faces

##### Attributes

Participants considered the vain-narcissist face as more narcissistic, selfish, conservative, and masculine (all *p*s < .001). The vain-narcissistic face was also rated as more vain and neurotic (all *p*s ≤ .002). Furthermore, the vain-narcissistic face was considered less kind, warm, likable, open, agreeable, and conscientious (all *p*s < .001). The largest effect sizes were found on narcissism (*d* = 1.42, 95% CI [1.23, 1.61]), vanity and selfishness (both *d* = 1.38, 95% CI [1.19, 1.57]). These patterns converge with what was found (with selfishness) in Experiment 1.

However, unlike patterns from Experiment 1, the vain-narcissistic (vs. non-vain) face was seen as more competent, successful, extraverted, and as having greater self-esteem (all *p*s < .001). We found no significant differences between the two faces on ratings of age (*p* = .021).

##### Values

There was a significant main effect of face type, *F*(1, 214) = 17.29, *p* < .001, $\eta_{p}^{2}$ = .075. Higher value importance ratings were attributed to the non-vain than the vain-narcissistic face. There was also a significant main effect of value type, *F*(2.75, 587.90) = 31.20, *p* < .001, $\eta_{p}^{2}$ = .127. Self-enhancement values were seen as more important than self-transcendence, openness, and conservation values (all *p*s < .005), with conservation seen as more important than self-transcendence values (*p* < .001).

These effects were qualified by a significant interaction, *F*(2.59, 553.64) = 153.71, *p* < .001, $\eta_{p}^{2}$ = .418. Replicating what was found with selfishness, the vain-narcissistic (vs. non-vain) face was perceived as valuing self-transcendence less (*p* < .001, $\eta_{p}^{2}$ = .512), and self-enhancement more (*p* < .001, $\eta_{p}^{2}$ = .516). The vain-narcissistic (vs. non-vain) face was also perceived as attaching less importance to openness (*p* = .002, $\eta_{p}^{2}$ = .043) and conservation values (*p* < .001, $\eta_{p}^{2}$ = .167).

##### Morality

The vain-narcissistic (vs. non-vain) face was judged as less moral (*p* < .001, Cohen’s *d* = −1.42). This replicates what was found with selfishness.

##### Workplace Roles

Replicating Experiment 1, the vain-narcissistic (vs. non-vain) face was judged as less suitable for a career in health services (*p* < .001, Cohen’s *d* = −0.85), and less desirable as a work colleague (*p* < .001, Cohen’s *d* = −0.92) and boss (*p* < .001, Cohen’s *d* = −0.71).

Unlike Experiment 1, the vain-narcissistic (vs. non-vain) face was judged as more suitable for a career in corporate management (*p* = .024, Cohen’s *d* = 0.53).

##### Leadership, Trustworthiness, Comfort with Physical Closeness, and Similarity

The results directly paralleled Experiment 1. Participants stated they were less likely to vote for the vain-narcissistic (vs. non-vain) face to be Prime Minister (*p* < .001, Cohen’s *d* = −0.35), and less likely to trust the vain-narcissistic (vs. non-vain) face to look after a loved one (*p* < .001, Cohen’s *d* = −0.92). Additionally, participants reported feeling less comfortable if trapped in an elevator with the vain-narcissistic (vs. non-vain) face (*p* < .001, Cohen’s *d* = −0.95) and reported lower similarity with the vain-narcissistic (vs. non-vain) face (*p* < .001, Cohen’s *d* = −0.49).

#### Associations Between Rater Narcissism and Evaluations of the Vain-narcissist

Consistent with narcissistic tolerance, greater rater narcissism was positively associated with perceived similarity with the vain-narcissistic face (*r*(213) = .19, *p* = .005), suggesting that narcissistic vanity, assessed indirectly via reverse correlation, facilitates effects of narcissistic tolerance. Rater narcissism was unrelated to perceptions of other outcomes (all *p*s > .030).

##### The Mediating Role of Perceived Similarity

**Figure 4.**
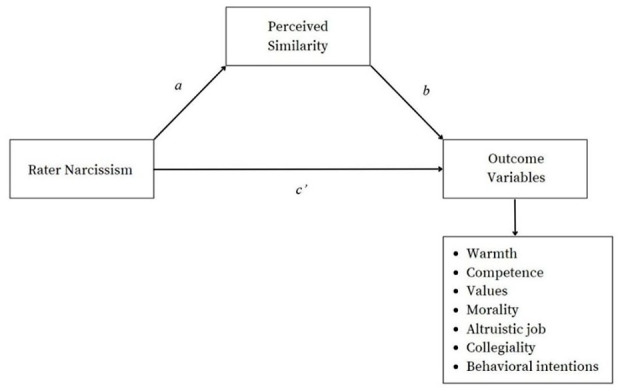
Conceptual framework illustrating tested indirect effects of rater narcissism on outcomes via perceived similarity. *Note. c*' = direct effect of X on Y; a*b = indirect effect of X on Y through perceived similarity.

We tested whether perceived similarity mediates the relationship between rater narcissism and evaluations of the vain-narcissistic face (see Figure 4 and Table 3). A sensitivity power analysis indicated that our sample size achieved 0.81 power at α = .05 for mediation models detecting indirect effects as small as Cohen’s *d* = 0.27 (Schoemann et al., 2017).

**Table 3.** Summary of Perceived Similarity Mediation Analyses: Vain-narcissist Face.

Outcome measure	Direct effect	Total effect	Indirect effect		Proportion Mediated Adjusted Index (%)
			Effect (Boot*SE*)	Bootstrap 95% CI	
Warmth	0.017 (0.056)	0.098 (0.061)	0.080* (0.030)	[0.026, 0.14]	82.30
Competence	−0.067 (0.069)	−0.014 (0.071)	0.053* (0.023)	[0.014, 0.10]	44.17
Values	1.59 (2.14)	3.10 (2.17)	1.51* (0.70)	[0.38, 3.08]	48.71
Morality	−0.089 (0.11)	0.089 (0.12)	0.17* (0.061)	[0.058, 0.30]	65.64
Altruistic job	0.039 (0.13)	0.085 (0.12)	0.046 (0.033)	[−0.0028, 0.13]	54.12
Collegiality	0.014 (0.079)	0.17 (0.094)	0.15* (0.055)	[0.049, 0.27]	91.43
Behavioral intentions	0.017 (0.068)	0.17 (0.085)	0.15* (0.050)	[0.051, 0.25]	89.83

* *p* < .05.

To minimize multiple testing, we conducted a factor analysis to assess the factor structure of the attribute items. The analyses revealed a two-factor structure that accounted for 64.52% of the total variance (Factor 1 = 41.54%; Factor 2 = 22.98%; see Table S2). Six items loaded onto the first factor “Warmth” (factor loadings of 0.52–0.90); four items loaded onto the second factor “Competence” (factor loadings of 0.49–0.74). Internal consistency of both factors was strong (both αs > .75), so we computed “Warmth” and “Competence” indices comprised of participants’ average item scores.

Our mediation analyses included the warmth and competences indices, as well as five outcome variables comprised of participants’ average scores on relevant items. “Values” represents perceptions of self-transcendent values relative to self-enhancement values. “Morality” represents perceptions of engaging in moral behaviors relative to immoral behaviors. “Altruistic job suitability” represents perceptions of suitability for altruistic (i.e., healthcare) relative to agentic (i.e., corporate management) job roles. “Collegiality” represents perceptions of desirability as a work colleague/boss. Finally, “Behavioral intentions,” combines perceptions of perceived leadership, trustworthiness, and comfort with physical closeness.

The models were tested using Hayes’ (2022) PROCESS model 4 (95% confidence intervals based on 10,000 bootstrap samples). Perceived narcissism did not directly predict any of the outcomes (all *p*s ≥ .336). However, significant indirect effects of rater narcissism via perceived similarity emerged for perceived warmth, competence, values, morality, collegiality, and behavioral intentions (but not for altruistic job suitability). Rater narcissism positively predicted similarity (*p* = .005), which in turn positively predicted six of the seven outcomes (all *p*s < .001). To better quantify these effects, Table 3 reports the Proportion Mediated (PM) Adjusted Index, which avoids overinflating estimates when proportion-mediated calculations are affected by small total effect sizes, as observed in some models (MacKinnon et al., 2000).

While our mediation models indicate indirect effects, via perceived similarity, they do not establish causality, particularly in the “b” paths (Bullock et al., 2010). While reversed models showed no significant indirect effects (see Table S3), this does not confirm directionality or eliminate confounding (Rohrer et al., 2022). The absence of direct effects suggests that confounding is less likely, though we acknowledge that power limitations could also contribute to the nonsignificant direct effects. Likewise, suppression would imply a reversed or strengthened predictor-outcome link when including the mediator, which was not observed. Nonetheless, we encourage future research using experimental or longitudinal designs to strengthen causal claims.

#### Comparing Selfish and Vain Classification Images

Next, we compared the selfish-narcissistic and non-selfish, and vain-narcissistic and non-vain faces via 2 (face type) × 2 (definition) mixed ANOVAs (see Table S4). Given that, unlike the selfish-narcissistic face, the vain-narcissistic face was perceived as more agentic (e.g., competent, successful, high self-esteem) relative to the non-vain face, we conducted pairwise comparisons between the selfish and vain-narcissistic faces, and between the non-selfish and non-vain faces, to examine the influence of vanity in eliciting different patterns of evaluations.

##### Attributes

The analyses revealed significant Face Type × Definition interactions on perceived narcissism, selfishness, vanity, masculinity, age, self-esteem, warmth, competence, success, openness, and extraversion (all *p*s < .001). The vain- (vs. selfish-) narcissistic face was seen as older, more narcissistic, selfish, vain, competent, successful, extraverted, less masculine, and as having greater self-esteem (all *p*s < .030). Furthermore, the non-vain (vs. non-selfish) face was seen as younger, more masculine, and less warm, competent, vain, successful, open, extraverted, and as having lower self-esteem (all *p*s < .019). We found no interaction effects for perceived political orientation, kindness, liking, conscientiousness, agreeableness, and neuroticism (all *p*s ≥ .064).

##### Values

For values, analyses revealed significant interactions for self-enhancement and openness. The vain- (vs. selfish-) narcissistic face was seen as more strongly endorsing self-enhancement values (*p* < .001). Further, the non-vain (vs. non-selfish) face was seen as less endorsing of self-enhancement and openness values (*p*s < .001). Non-significant interactions were found for self-transcendence and conservation values (both *p*s ≥ .087).

##### Morality

For morality, face type did not significantly interact with definition to influence ratings (*p* = .294).

##### Workplace Roles

Regarding occupational suitability, interactions were found for both corporate management and health services roles (*p*s < .005). The vain- (vs. selfish-) narcissistic face was seen as more suitable for a corporate management role (*p* < .001), with the non-vain (vs. non-selfish) face seen as less suitable for corporate management and health services careers (*p*s < .001).

For workplace relations, the analyses revealed a significant interaction on ratings of the faces’ desirability as bosses (*p* = .009), but not colleagues (*p* = .775). The non-vain (vs. non-selfish) face was seen as a less desirable boss (*p* < .001).

##### Behavioral Intentions

Here, analyses revealed significant interactions on ratings of voting intentions and trust (all ps ≥ .028) but not comfort in close physical proximity (*p* = .414). The non-vain (vs. non-selfish) face was ascribed both lower voting intentions and trust ratings (*p*s < .001).

##### Similarity

Finally, face type interacted with definition on perceived similarity ratings (*p* = .004). Participants reported sharing less in common with the non-vain (vs. non-selfish) face (*p* < .001).

### Discussion

Experiment 2 examined visual representations of vain-narcissistic and non-vain faces and tested the consequences of these representations. Overall, the vain-narcissistic (vs. non-vain) face was perceived less favorably (e.g., as more narcissistic, selfish, self-enhancing, and as less warm, likable, and kind). However, whereas Experiment 1’s selfish-narcissistic (vs. non-selfish) face was seen as relatively lacking in agentic traits (e.g., competence, success, extraversion, self-esteem), we found contrasting results in Experiment 2. Specifically, the vain-narcissistic face was seen as more competent, successful, extraverted, suitable for corporate management, and higher in self-esteem than its non-vain counterpart.

Exploratory comparisons between (a) the selfish-narcissistic and vain-narcissistic and (b) non-selfish and non-vain faces further supported the notion that highlighting the vanity component of narcissism prompts greater inferences of agency. The vain- (vs. selfish-) narcissistic face was seen as older, more narcissistic, selfish, vain, competent, successful, extraverted, as having higher self-esteem, more greatly endorsing self-enhancement values, and as more suitable for a career in corporate management.

Furthermore, we observed different patterns of associations between rater narcissism and evaluations of the selfish- versus vain-narcissistic faces. In Experiment 1, rater narcissism was positively associated with pejorative evaluations of the selfish-narcissist. However, in Experiment 2, rater narcissism positively correlated with greater perceived similarity with the vain-narcissist, suggesting that vanity plays a crucial role in facilitating the narcissism-similarity link, which subsequently predicted favorable outcomes (e.g., warmth, competence, morality).

In Experiments 1 and 2, the narcissistic faces were evaluated relative to a different non-narcissist. As such, contrast effects may have influenced the relative nature of participants’ judgments. Further, while our findings suggest that narcissistic vanity is important in eliciting multifaceted and more favorable perceptions of narcissists, as well as bolstering narcissistic tolerance via perceived similarity, it does not explain why. One possibility is that narcissistic vanity implies physically attractive features, promoting the impression of more desirable traits and the narcissism-similarity link. Indeed, attractive people are perceived to be high in vanity (Han & Laurent, 2023). Given these results, Experiment 3 focuses on perceptions of the facial images in the domain of sexual/romantic attraction.

## Experiment 3—Do People Find Narcissistic (vs. Non-narcissistic) Faces Attractive?

Experiment 3 examined perceptions of the selfish- and vain-narcissistic *and* non-selfish and non-vain images on dimensions of physical attraction and sexual/romantic partnership. Understanding such perceptions is important because narcissists demonstrate distinct qualities in their romantic relationships (Foster & Brunell, 2018), putting greater effort into their appearance and being considered attractive by others at the first meeting (Holtzman & Strube, 2013). Yet, over time, narcissism elicits both self- and partner-reported relationship dissatisfaction and diminished long-term commitment (Jonason & Buss, 2012).

Because narcissism represents a double-edged sword in the context of romance and attraction, we were interested in perceptions of narcissistic and non-narcissistic faces on these dimensions. In Experiment 3, we focused on five facets relevant to romantic perceptions: attraction, suitability for short-term partnership, suitability for long-term partnership, friendship, and toxic relationship behaviors—dimensions linked to narcissism (Holtzman & Strube, 2013; Jauk et al., 2021).

We once again focused on evaluations of perceived similarity. As proposed by the similarity-attraction hypothesis, individuals experience greater attraction to people like themselves (Montoya & Horton, 2013). Studies of this effect have highlighted the importance of *perceived*, rather than *actual*, similarity in predicting romantic attraction (Tidwell et al., 2013). Further, we measured perceived familiarity, given its reliability as a predictor of attraction (Reis et al., 2011).

Finally, like Experiments 1 and 2, we focused on evaluations of warmth, competence, masculinity, and narcissism. Comparing the narcissistic images, Experiment 2 found that people perceived the vain- (vs. selfish-) narcissistic face as more narcissistic; in Experiment 3 we tested whether this effect would replicate. We also asked participants to indicate the extent to which they would secretly enjoy being each of the faces. This exploratory item assessed whether certain faces were seen as more appealing.

We predicted that the narcissistic faces would generally be perceived less favorably than the non-narcissistic faces. However, we explored whether the selfish vs. vain differentiation would elicit distinct judgments of attraction and suitability for friendship and short- and long-term partnerships. Given that the vain- (vs. selfish-) narcissistic face was perceived more favorably, we were keen to examine whether this effect would carry over to romantic perceptions.

### Method

#### Participants

We recruited 202 UK participants through Prolific (101 females, 99 males, 2 prefer not to say; *M*_age_ = 38.06, *SD*_age_ = 12.71; see Table S5 for further details).

An a priori power analysis (G*Power 3.1; Faul et al, 2009) using the “ANOVA: Repeated measures, within factors” method suggested that 138 participants were required to ensure 80% statistical power for a small effect size (*f* = 0.10). We conducted an additional a priori power analysis to determine the sample size needed to achieve enough power (80%) to detect a small to moderate effect size (*r* = .20; *p* < .05; two tailed) for correlations between individual difference measures and face evaluations. Results indicated that a sample of 193 was sufficient.

#### Materials and Procedure

##### Face Rating Task

**Figure 5.**
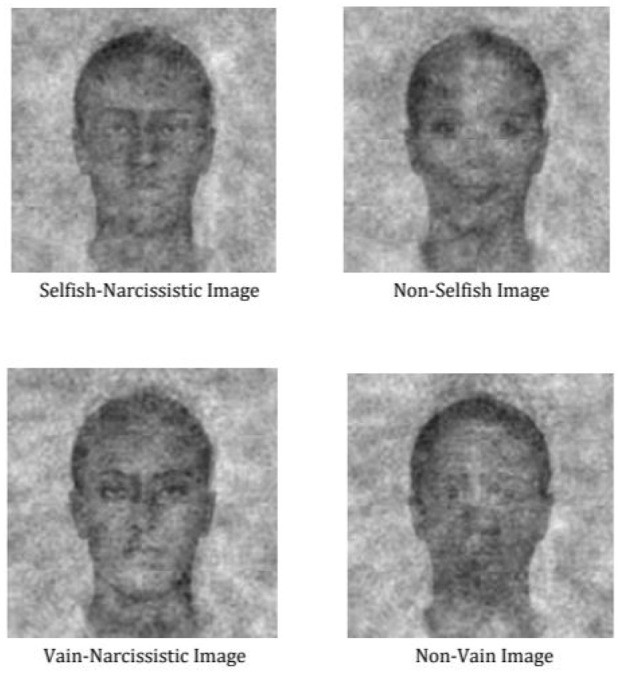
Narcissistic and non-narcissistic facial images.

Participants completed the task via Qualtrics. After providing consent, participants made judgments about the faces (Figure 5) on various dimensions. As in Experiments 1 and 2, participants evaluated each face individually on a separate screen, and no information was provided about the faces or how they were generated.

First, participants evaluated the faces on a series of dimensions (presented in a random order). These comprised measures of friend value (“To what extent would you desire this person as a platonic friend?”), desirability as a short-term partner (“To what extent would you, personally, desire this person for a short-term sexual encounter [e.g., a one-night stand?]”), and long-term partner (“To what extent would you, personally desire this person for a long-term committed relationship [e.g., to marry, raise children with, etc.?]”) (from Rauthmann & Kolar, 2013), and perceived attractiveness (“To what extent do you, personally, find this person physically attractive?”). For the latter three questions, participants also indicated how much they thought that people, *in general*, would find the faces attractive and desirable as a short-/long-term partner. We included these *general* perspectives to mitigate against potential effects of participant gender, sexual orientation, and/or relationship status on appraisals of perceived personal attraction and sexual/romantic desirability.

Additionally, these dimensions included perceived toxic relationship behaviors, adapted from Frederick and Haselton (2007). Participants were asked “How likely is it that this person”: (a) has a bad temper; (b) would ignore their partner’s emotional needs; (c) would be abusive to their partner; and (d) would be unfaithful to their partner. We also measured participants’ perceptions of the faces’ perceived warmth, competence and masculinity (“How X does this person look?”), perceived familiarity (“To what extent does this person feel familiar to you?”), and a three-item measure of perceived similarity (from Burton et al., 2017).

Next, we presented participants with each face in a random order and asked “Secretly, how much would you enjoy being this person?” followed by “How narcissistic does this person look?” Perceived narcissism was included last to ensure that the concept of narcissism was not made salient prior to participants’ evaluations. Across all dimensions, participants responded on seven-point scales (1 = *Not at all*, 7 = *Extremely*).

Following this task, participants completed the SINS (*M* = 2.09, *SD* = 1.33). Participants also completed several additional individual differences measures, presented in random order, that were included for exploratory purposes and not reported below. These measures, and their relationship to evaluations of all four faces, can be found in Tables S6 to S29. Finally, participants completed demographic information.

### Results

We begin by presenting our preregistered testing for relative differences between the faces. We conducted 2 (face type) × 2 (definition) repeated measures ANOVAs testing for differences between the narcissistic and non-narcissistic faces, the selfish and vain faces, and their interaction, on ratings of outcome variables. Significant main and interaction effects were interpreted via Bonferroni corrected pairwise comparisons. Descriptive statistics for each face, along with their absolute differences on all ratings, are presented in Table 4. For parsimony, we focus on comparing (a) the Narcissistic and Non-narcissistic Faces, (b) the Vain- (vs. Selfish-) narcissistic Faces, and (c) the Non-vain (vs. Non-Selfish) faces. Other analyses are presented in Supplemental Material 8.

**Table 4.** Descriptive Statistics and ANOVA Results.

	Narcissist		Non-narcissist		Repeated measures ANOVA			
Outcome measure	Selfish^a^	Vain^b^	Selfish^c^	Vain^d^	Predictor	*F*	$\eta_{p}^{2}$	*p*
Friend	2.52**,^bcd^	2.23**,^acd^	3.92^abd^	3.00**,^abc^	Face	163.22	.448	<.001
					Definition	89.59	.308	<.001
					Face × Definition	21.95	.098	<.001
Attraction (G)	3.12**,^cd^	3.17**,^cd^	4.15^abd^	2.83**,^abc^	Face	20.38	.092	<.001
					Definition	109.90	.353	<.001
					Face × Definition	109.52	.353	<.001
Attraction (P)	1.64**,^bc^	1.91**,^acd^	3.26**,^abd^	1.64**,^bc^	Face	90.66	.311	<.001
					Definition	123.56	.381	<.001
					Face × Definition	149.77	.427	<.001
Short-term partner (G)	3.05**,^cd^	3.25**,^cd^	4.18^abd^	2.55**,^abc^	Face	6.94	.033	.009
					Definition	100.38	.333	<.001
					Face × Definition	158.35	.441	<.001
Short-term partner (P)	1.43**,^bc^	1.65**,^acd^	2.33**,^abd^	1.40**,^bc^	Face	21.27	.096	<.001
					Definition	28.84	.125	<.001
					Face × Definition	54.54	.213	<.001
Long-term partner (G)	2.81**,^c^	2.89**,^c^	4.41**,^abd^	3.01**,^c^	Face	118.62	.371	<.001
					Definition	94.16	.319	<.001
					Face × Definition	105.40	.344	<.001
Long-term partner (P)	1.50**,^cd^	1.50**,^cd^	2.49**,^abd^	1.73**,^abc^	Face	71.87	.263	<.001
					Definition	34.75	.147	<.001
					Face × Definition	23.17	.103	<.001
Toxic behaviors	4.38**,^cd^	4.28*^cd^	2.78**,^abd^	3.04**,^abc^	Face	278.23	.581	<.001
					Definition	2.34	.012	.127
					Face × Definition	11.04	.052	<.001
Familiar	2.07**,^c^	1.94**,^cd^	2.78**,^abd^	2.30**,^bc^	Face	46.64	.188	<.001
					Definition	23.23	.104	<.001
					Face × Definition	7.65	.037	.006
Similar	2.58**,^cd^	2.55**,^cd^	3.56**,^abd^	2.82**,^abc^	Face	86.00	.300	<.001
					Definition	48.28	.194	<.001
					Face × Definition	40.07	.166	<.001
Warm	1.93**,^cd^	1.91**,^cd^	5.02**,^ab^	3.03**,^ab^	Face	552.69	.733	<.001
					Definition	184.59	.479	<.001
					Face × Definition	195.84	.494	<.001
Competent	3.40**,^c^	3.60**,^cd^	4.36**,^abd^	3.33**,^bc^	Face	20.25	.092	<.001
					Definition	62.38	.237	<.001
					Face × Definition	80.96	.287	<.001
Masculine	5.23**,^bcd^	4.15^ac^	1.88**,^abd^	4.50**,^ad^	Face	380.00	.654	<.001
					Definition	135.26	.402	<.001
					Face × Definition	359.61	.641	<.001
Secret	1.94**,^c^	1.98**,^c^	3.23**,^abd^	2.05**,^c^	Face	61.16	.233	<.001
					Definition	85.73	.299	<.001
					Face × Definition	70.31	.259	<.001
Narcissistic	4.15^bcd^	4.94**,^acd^	2.63**,^ab^	2.49**,^ab^	Face	249.51	.554	<.001
					Definition	19.33	.088	<.001
					Face × Definition	31.68	.136	<.001

*Note*. Superscripts with a different letter differ at *p* < .05.

** *p* < .05 difference from scale midpoint.

We then report our preregistered testing for associations between rater narcissism and evaluations of the narcissistic and non-narcissistic faces’ perceived similarity and familiarity. Finally, we report additional post-hoc exploratory testing of the mediating role of perceived similarity and familiarity on the relationship between rater narcissism and romantic perceptions of the vain-narcissistic face.

#### Comparing the Narcissistic and Non-narcissistic Faces

Overall, the narcissistic (vs. non-narcissistic) faces were seen as less attractive (general and personal), less suitable for platonic friendship, short- and long-term partnerships (general and personal), and as more likely to engage in toxic relationship behaviors (*p*s ≤ .009). The narcissistic (vs. non-narcissistic) faces were also ascribed lower similarity, familiarity, warmth, competence, secret enjoyment scores, and judged as more masculine, and narcissistic (*p*s < .001).

#### Comparing the Vain- (vs. Selfish-) narcissistic Faces

Comparing between the two narcissistic faces, the vain- (vs. selfish-) narcissist was seen as less suitable for friendship, but as more *personally* physically attractive and *personally* suitable for short-term partnership (*p*s ≤ .017). The vain- (vs. selfish-) narcissist was also seen as less masculine and more narcissistic (*p*s < .001). No differences emerged on other variables (*p*s ≥ .072).

#### Comparing the Non-vain (vs. Non-selfish) Faces

Comparing between the two non-narcissistic faces, the non-vain (vs. non-selfish) face was seen as less attractive (general and personal), less suitable for platonic friendship and short- and long-term partnerships (general and personal), and as more likely to engage in toxic relationship behaviors (*p*s ≤ .006). The non-vain (vs. non-selfish) face was also ascribed lower similarity, familiarity, warmth, competence, and secret enjoyment scores (*p*s < .001). The non-vain (vs. non-selfish) non-narcissist was seen as more masculine (*p* < .001), but no more or less narcissistic (*p* = 1.00).

#### Summary

The results broadly support our hypothesis that the narcissist (vs. non-narcissist) faces would be perceived less favorably in the context of sexual/romantic attraction. That said, we found a number of meaningful interactions. In line with Experiment 2, the vain- (vs. selfish-) narcissistic face was judged as more desirable (e.g., more personally attractive and suitable for short-term partnership) and the non-vain (vs. non-selfish) face was judged as less desirable (e.g., less attractive). This further supports the notion that emphasizing the vanity aspect of narcissism elicits distinct (and more favorable) evaluations of narcissists.

#### Associations Between Rater Narcissism and Perceptions of Similarity and Familiarity

We tested associations between raters’ self-reported narcissism and perceptions of perceived similarity and familiarity with the narcissistic faces via Bonferroni corrected Pearson’s correlations.

Replicating Experiments 1 and 2, rater narcissism was significantly associated with perceived similarity with the vain-narcissist (*r*(202) = .19, *p* = .008), but not the selfish-narcissist (*r*(202) = .07, *p* = .321). Similarly, rater narcissism was significantly associated with perceived familiarity with the vain-narcissist (*r*(202) = .22, *p* = .002), but not the selfish-narcissist (*r*(202) = .13, *p* = .065).

#### The Mediating Roles of Perceived Similarity and Familiarity with the Vain-narcissistic Face

To explore the association between rater narcissism and perceived similarity and familiarity with the vain-narcissist, we tested whether perceived similarity and familiarity mediated the relationship between rater narcissism and evaluations of the face’s sexual/romantic suitability. This was done using Hayes’ (2022) PROCESS model 4 (95% confidence intervals based on 10,000 bootstrap samples).

The model predictor was rater narcissism, and the mediators were perceived similarly and perceived familiarity. The two outcome variables tested were “sexual/romantic suitability,” which was an index created using participants’ average scores on perceived attraction, suitability for short- and long-term partnerships (general and personal), and suitability for friendship (α = .84) and toxic relationship behaviors.

We found significant indirect effects of rater narcissism on sexual/romantic suitability via both perceived similarity (*b* = 0.030, *SE* = 0.014, 95% CI [0.0053, 0.059]) and familiarity (*b* = 0.059, *SE* = 0.023, 95% CI [0.021, 0.11]). Rater narcissism did not directly predict sexual/romantic suitability (*b* = −0.048, *SE* = 0.039, *t* = −1.24, *p* = .217). Using the PM Adjusted Index, perceived similarity and familiarity mediated 64.78% of the positive relationship between rater narcissism and perceptions of greater sexual/romantic suitability.

For toxic relationship behaviors, the indirect of effect of rater narcissism via perceived similarity was significant (*b* = −0.066, *SE* = 0.029, 95% CI [−0.13, −0.013]), while the indirect effect via perceived familiarity was nonsignificant (*b* = −0.0042, *SE* = 0.015, 95% CI [−0.036, 0.025]). As with sexual/romantic suitability, rater narcissism did not significantly directly predict perceptions of toxic relationship behaviors (*b* = 0.066, *SE* = 0.066, *t* = 0.99, *p* = .324). Using the PM Adjusted Index, perceived similarity mediated 50.27% of the negative relationship between rater narcissism and perceptions of toxic relationship behaviors.

### Discussion

Building upon Experiments 1 and 2, Experiment 3 explored romantic perceptions of selfish- and vain-narcissistic and non-selfish and non-vain faces. Overall, the narcissistic (vs. non-narcissistic) faces were seen as less suitable for friendship and romantic partnership (short- and long-term), less attractive, and as more likely to engage in toxic relationship behaviors. They were also seen as less warm, competent, familiar, similar, and as more narcissistic. However, consistent with the comparison of Experiments 1 and 2, the vain-narcissist was more romantically favored relative to the selfish-narcissist. Further, the non-selfish face was perceived more favorably than the non-vain face. Thus, highlighting the vanity aspect of narcissism prompts greater interference of agentic traits and also elicits more favorable judgments regarding romance and attraction.

Finally, replicating the comparison of Experiments 1 and 2, the vain-narcissist was seen as significantly more narcissistic relative to the selfish-narcissist. That this effect was found when the faces were rated separately (Experiment 2) *or* together (Experiment 3) is noteworthy, suggesting that vanity, along with selfishness tendencies, is fundamental to lay conceptualizations of narcissism.

## General Discussion

Judging people based on their facial features influences our daily interactions and decisions. While previous research has focused on individuals’ ability to detect facially signaled narcissism (Alper et al., 2021; Holtzman, 2011) or physical manifestations of narcissism (Giacomin & Rule, 2019), we adopted a novel and theoretically based perspective: visual representations of narcissists and their consequences. Based on conceptual models showing that people view narcissism in relation to entitlement/antagonism (i.e., selfishness) and grandiosity (i.e., vanity), we utilized a bottom-up approach to generate faces prototypical of both these dimensions (and their non-narcissistic counterparts). Subsequently, three naïve samples rated these faces on personal attributes, values, and behaviors (Experiments 1 and 2) and perceived attractiveness and romantic suitability (Experiment 3).

While narcissistic (vs. non-narcissistic) faces were broadly perceived unfavorably, the vain- (vs. selfish-) narcissist was seen as more agentic and suitable for romantic partnership, suggesting that the inclusion of vanity has positive interpersonal outcomes. Indeed, previous research has linked narcissistic vanity with increased popularity (Back et al., 2010). Relatedly, when evaluating narcissistic targets/traits in the absence of physical appearance cues, participants tend to demonstrate particularly negative perceptions (Hart & Adams, 2014), suggesting that the inclusion of vanity within narcissism elicits a more positive conceptualization of what it means to be narcissistic.

Importantly, rater narcissism was positively associated with perceived similarity with the vain- (but not selfish-) narcissistic face, suggesting that inferences of vanity are crucial in fostering the narcissism-similarity link. Furthermore, this link mediated favorable impressions of the vain-narcissist (e.g., warmth, competence, leadership qualities) and increased perceptions of their attraction and romantic suitability. This extends our knowledge of narcissistic tolerance in several ways. First, our findings demonstrate that narcissistic tolerance can be replicated via facially communicated narcissism, even when overt aspects of narcissism remain undisclosed. Previously, narcissistic tolerance had only been observed when narcissistic raters were exposed to explicit expressions of narcissistic traits (Burton et al., 2017; Hart & Adams, 2014).

Second, our findings highlight the importance of narcissistic vanity in supporting narcissistic tolerance. Notably, however, our research focused on the effects of narcissistic tolerance of grandiose (i.e., vain) expressions of narcissism from individuals scoring high on grandiose measures of the traits (i.e., NPI/SINS score). Future research may investigate whether highlighting antagonistic aspects of narcissism (e.g., selfishness) might heighten the effect of narcissistic tolerance among individuals high in antagonistic narcissism.

Third, we found that narcissistic tolerance is largely mediated via perceived similarity. This demonstrates that the effects of narcissistic tolerance, underpinned by perceived similarity, can manifest across multiple domains (e.g., perceived values, career suitability, attraction) via faces. This may represent an instantiation of false consensus, whereby narcissistic individuals perceive vain narcissists as sharing their own attributes and values (see Marks & Miller, 1987). Which particular factors drive and affect similarity perceptions represents a worthy endeavor for future investigations.

### Limitations and Future Directions

There are some limitations of the present research. First, we focused on participants’ visual representations of two core facets of narcissism—selfishness and vanity—because of their prominence in how people define narcissism (Smith et al., 2024). Future research could consider how people mentally represent other dimensions of narcissism, such as vulnerable narcissism. Second, as our designs were cross-sectional, future research could more directly test causal pathways in our mediation models. Third, we did not collect data from generators or raters about their race. Future research might consider assessing such data, given findings on cross-race face perception (Singh et al., 2022). Fourth, our classification images reflect public perceptions rather than the facial structures of individuals high in trait narcissism. Comparing our images to Faceaurus (Holtzman, 2011), a dataset of composite faces derived from individuals high versus low in various traits, could help evaluate whether perceived and actual facial features align.^1^ Fifth, our generators were university students. Future research might assess how more diverse adult samples mentally represent selfish and vain narcissists. That said, research has demonstrated that lay conceptualizations of narcissism are relatively stable across age (Smith et al., 2024). Similarly, our samples were from a WEIRD nation (Henrich et al., 2010). Future research could explore visual representations of narcissism cross-culturally. Research has demonstrated cross-cultural differences in levels of narcissism (Fatfouta et al., 2021). Given these differences, and cross-cultural differences in how facial areas are used to perceive expressions (e.g., Jack et al., 2012), future research could address potential differences in representations of narcissism across cultures.

### Concluding Summary

Use of the term narcissist has infiltrated the cultural zeitgeist. Across three experiments, we demonstrate that observing the image of a shared representation of a narcissistic face drives meaningful interpersonal inferences and social outcomes, even when that representation is purposefully isolated from information that might link it with narcissism. These outcomes are distinctly predicted by the aspect of narcissism emphasized when generating these representations, with the vain- (vs. selfish-) narcissistic face generally perceived more favorably. Further, we demonstrated the effects of narcissistic tolerance using a novel method, across multiple measures (e.g., NPI, SINS) and outcome variables (e.g., workplace perceptions, political leadership, sexual/romantic attraction), bolstering the generality of narcissistic tolerance effects.

## Supplemental Material

sj-docx-1-psp-10.1177_01461672251339014 – Supplemental material for What Narcissists Look Like and Why It’s Important Supplemental material, sj-docx-1-psp-10.1177_01461672251339014 for What Narcissists Look Like and Why It’s Important by Sarah Smith, Travis Proulx and Geoffrey Haddock in Personality and Social Psychology Bulletin

## Notes

1. We thank a reviewer for highlighting this interesting point.
