# Supplementary material for: What Narcissists Look Like and Why It’s Important
Source: Pers Soc Psychol Bull. 2025 May 22;52(8):2455–72. doi: 10.1177/01461672251339014 (PMC13310266; doi:10.1177/01461672251339014)
Supplement: sj-docx-1-psp-10.1177_01461672251339014 – Supplemental material for What Narcissists Look Like and Why It’s Important [file sj-docx-1-psp-10.1177_01461672251339014.docx]

Supplementary Materials

Contents

[Supplementary Materials 1: Classification Images Generated by High (vs. Low) Scorers Across Four Narcissism Scales (Experiment 1) 3](#_Toc180745829)

[**Figure S1** 3](#_Toc180745830)

[**Figure S2** 4](#_Toc180745831)

[**Figure S3** 5](#_Toc180745832)

[**Figure S4** 6](#_Toc180745833)

[Supplementary Materials 2: Comparing Non-Narcissistic (vs. Selfless) Faces (Experiment 1) 7](#_Toc180745834)

[**Figure S5** 7](#_Toc180745835)

[**Table S1** 8](#_Toc180745836)

[Supplementary Materials 3: Factor Loadings for Attribute Items (Experiment 2) 9](#_Toc180745837)

[**Table S2** 9](#_Toc180745838)

[Supplementary Materials 4: Alternative Mediation Models (Experiment 2) 10](#_Toc180745839)

[**Table S3** 10](#_Toc180745840)

[Supplementary Materials 5: Mixed ANOVA Results (Experiment 2) 11](#_Toc180745841)

[**Table S4** 11](#_Toc180745842)

[Supplementary Materials 6: Sample Demographics (Experiment 3) 14](#_Toc180745843)

[**Table S5** 14](#_Toc180745844)

[Supplementary Materials 7: Associations Between Additional Experiment Measures and Evaluations of the Four Faces (Experiment 3) 15](#_Toc180745845)

[**Table S6** 15](#_Toc180745846)

[**Table S7** 17](#_Toc180745847)

[**Table S8** 18](#_Toc180745848)

[**Table S9** 19](#_Toc180745849)

[**Table S10** 20](#_Toc180745850)

[**Table S11** 21](#_Toc180745851)

[**Table S12** 22](#_Toc180745852)

[**Table S13** 23](#_Toc180745853)

[**Table S14** 24](#_Toc180745854)

[**Table S15** 25](#_Toc180745855)

[**Table S16** 26](#_Toc180745856)

[**Table S17** 27](#_Toc180745857)

[**Table S18** 28](#_Toc180745858)

[**Table S19** 29](#_Toc180745859)

[**Table S20** 30](#_Toc180745860)

[**Table S21** 31](#_Toc180745861)

[**Table S22** 32](#_Toc180745862)

[**Table S23** 33](#_Toc180745863)

[**Table S24** 34](#_Toc180745864)

[**Table S25** 35](#_Toc180745865)

[**Table S26** 36](#_Toc180745866)

[**Table S27** 37](#_Toc180745867)

[**Table S28** 38](#_Toc180745868)

[**Table S29** 39](#_Toc180745869)

[Supplementary Materials 8: Reporting ANOVA Results of Additional Face Comparisons (Experiment 3) 40](#_Toc180745870)

[References 42](#_Toc180745871)

# Supplementary Materials 1: Classification Images Generated by High (vs. Low) Scorers Across Four Narcissism Scales (Experiment 1)

## **Figure S1**

*Average Classification Images of Narcissist and Non-Narcissist Generated by High (vs. Low) NPI Scorers*


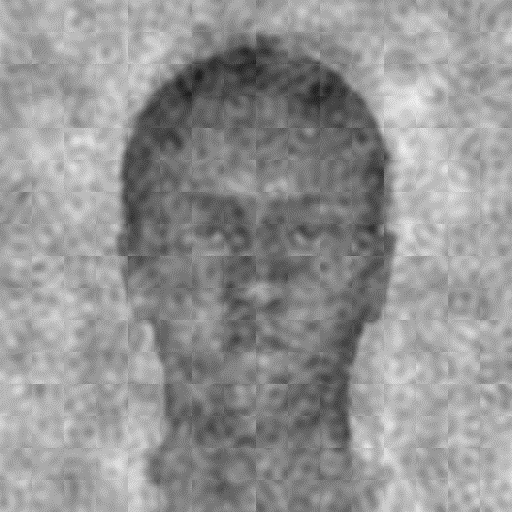

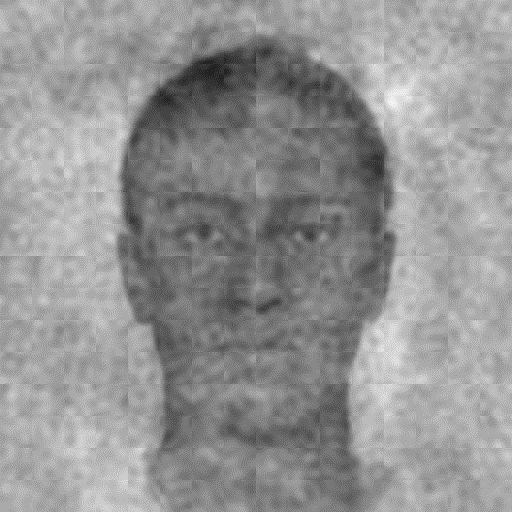


Narcissistic Face (High NPI) Narcissistic Face (Low NPI)


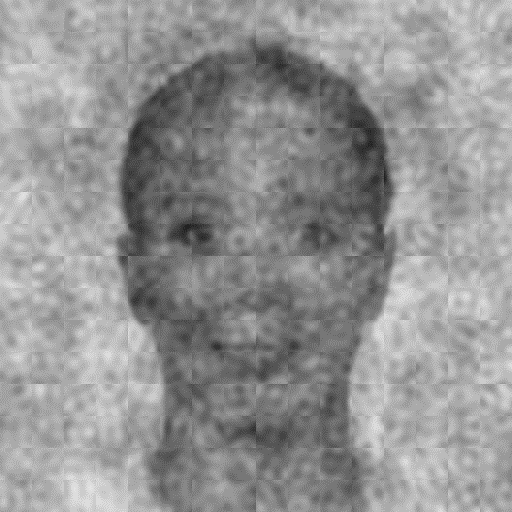

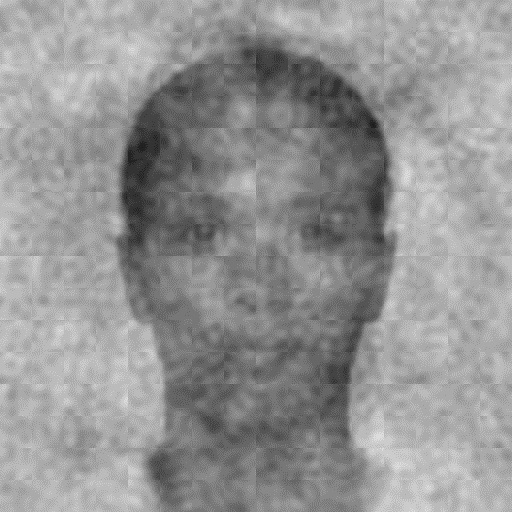


Non-Narcissistic Face (High NPI) Non-Narcissistic Face (Low NPI)

## **Figure S2**

*Average Classification Images of Narcissist and Non-Narcissist Generated by High (vs. Low) Narcissistic Admiration and Rivalry (NARQ) Scorers*


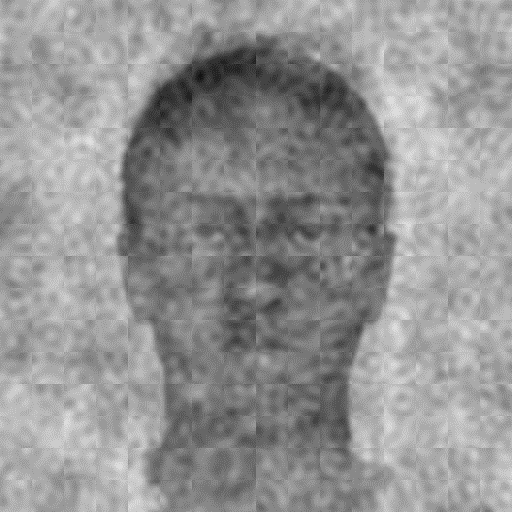

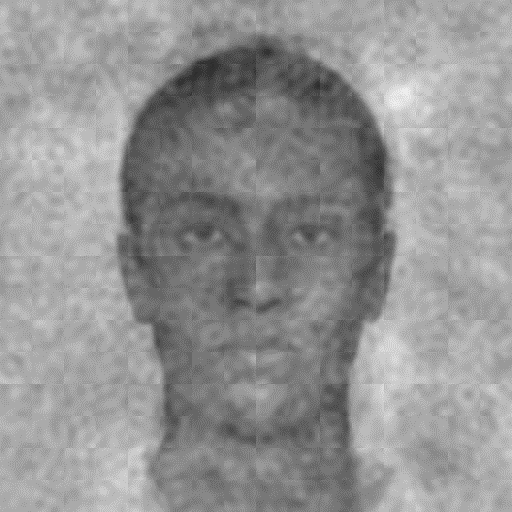


Narcissistic Face (High NARQ) Narcissistic Face (Low NARQ)


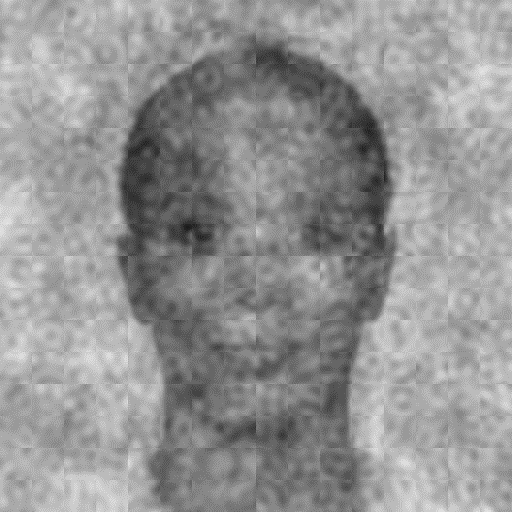

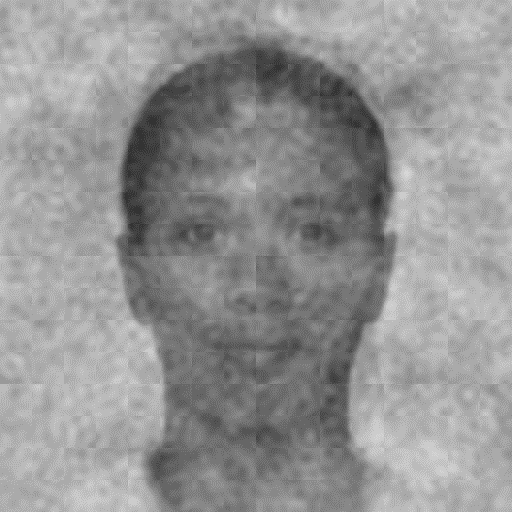


Non-Narcissistic Face (High NARQ) Non-Narcissistic Face (Low NARQ)

## **Figure S3**

*Average Classification Images of Narcissist and Non-Narcissist Generated by High (vs. Low) Five-Factor Narcissism Inventory Vulnerable Narcissism (FFNI-VN) Scorers*


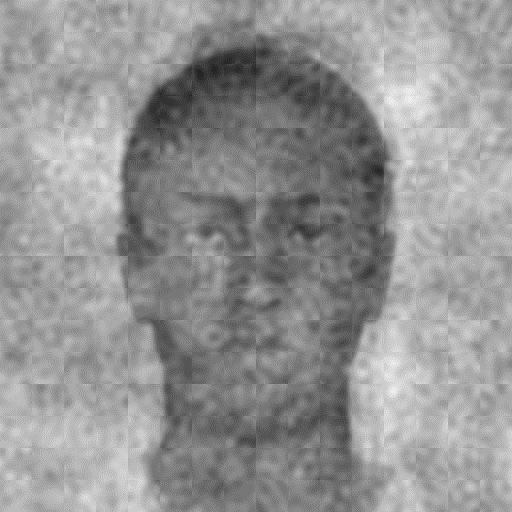

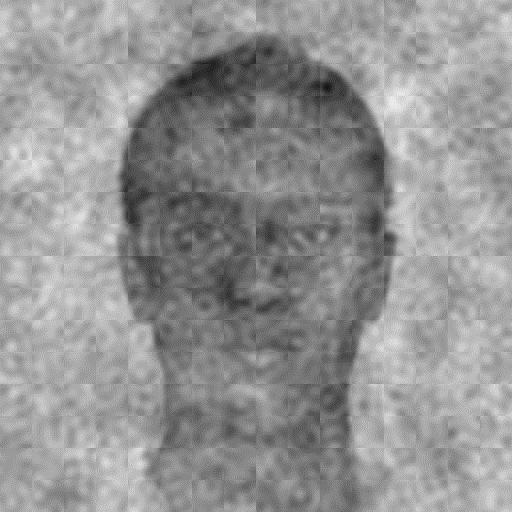


Narcissistic Face (High FFNI-VN) Narcissistic Face (Low FFNI-VN)


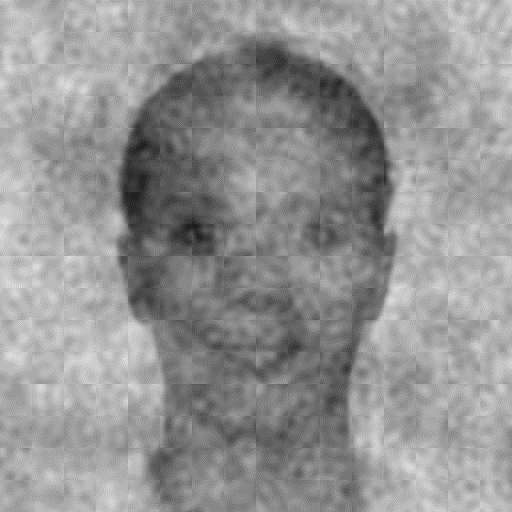

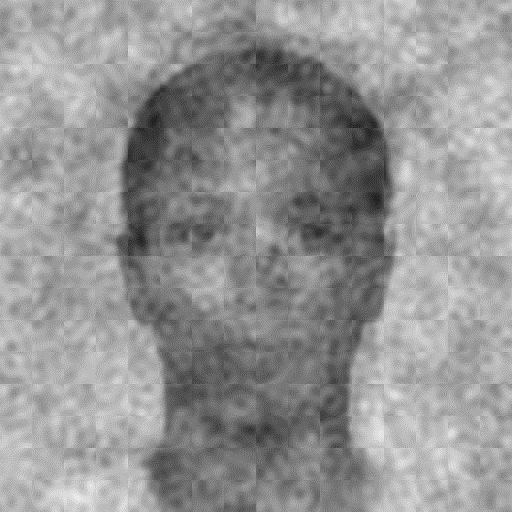


Non-Narcissistic Face (High FFNI-VN) Non-Narcissistic Face (Low FFNI-VN)

## **Figure S4**

*Average Classification Images of Narcissist and Non-Narcissist Generated by High (vs. Low) Communal Narcissism Inventory (CNI) Scorers*


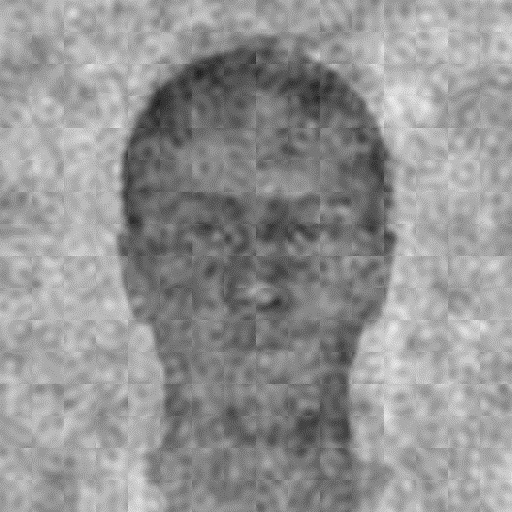

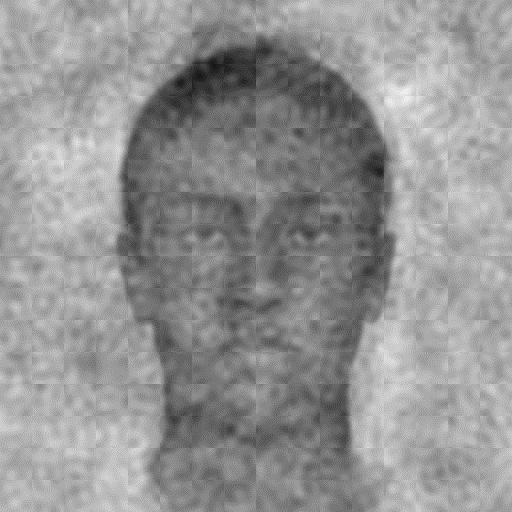


Narcissistic Face (High CNI) Narcissistic Face (Low CNI)


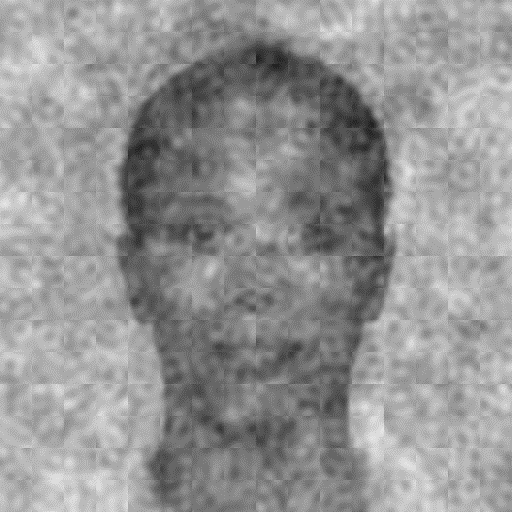

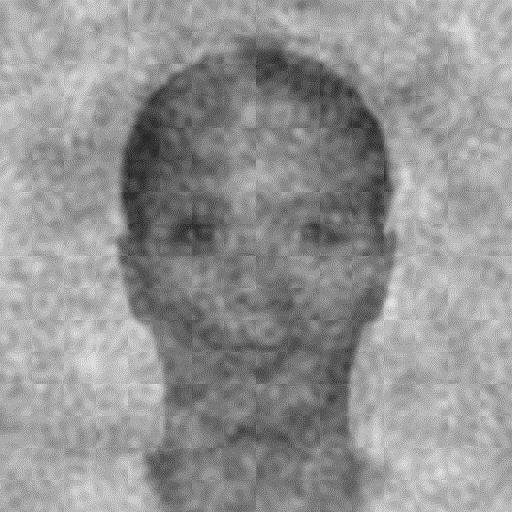


Non-Narcissistic Face (High CNI) Non-Narcissistic Face (Low CNI)

# Supplementary Materials 2: Comparing Non-Narcissistic (vs. Selfless) Faces (Experiment 1)

To ensure that the faces not selected as narcissistic (i.e., the non-narcissistic face) sufficiently approximated a selfless face (see Figure 5), we conducted a separate pilot Experiment (*N* = 264). Here, we tested for relative differences between the two faces using Bonferroni corrected independent samples *t*-tests. As shown in Table S1, we found no differences in ratings between the two faces (all *p*s > .058) other than on perceptions of age; the non-narcissistic face was seen as older (*p* < .001).

## **Figure S5**

*Average Classification Images of Non-Narcissistic (vs. Selfless) Faces*


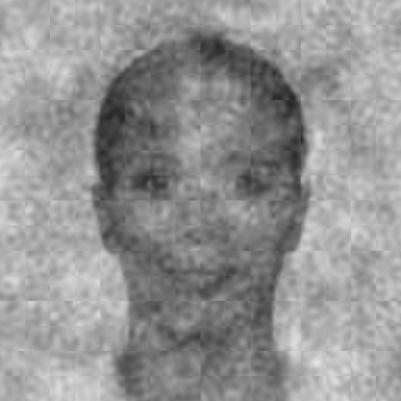

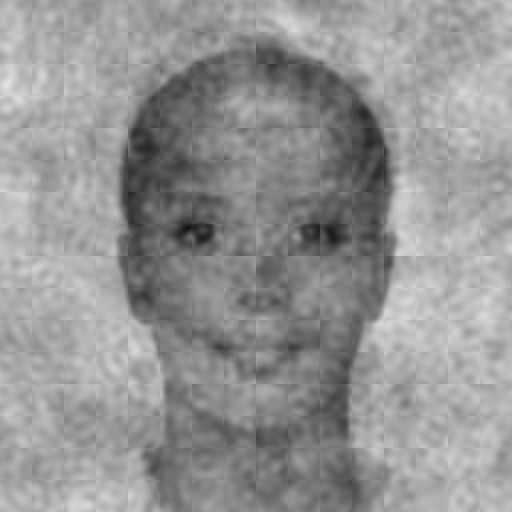


Non-Narcissistic Face Selfless Face

## **Table S1**

*Comparing Ratings of Non-Narcissistic and Selfless Faces On Attributes*

|  | Non-narcissistic | | Selfless | |  |  |  |
| --- | --- | --- | --- | --- | --- | --- | --- |
|  | *M* | *SD* | *M* | SD | *t* | *p* | *Cohen’s d* |
| Narcissistic | 3.45 | 1.51 | 3.23 | 1.50 | -0.10 | .320 | -0.01 |
| Masculine | 2.03 | 1.13 | 1.93 | 0.96 | -0.68 | .500 | -0.08 |
| Kind | 5.32 | 1.13 | 5.58 | 1.08 | 1.62 | .106 | 0.20 |
| Selfish | 3.07 | 1.36 | 2.86 | 1.30 | -1.09 | .277 | -0.13 |
| Vain | 4.05 | 1.51 | 3.68 | 1.38 | -1.79 | .075 | -0.22 |
| Self-Esteem | 4.87 | 1.17 | 5.10 | 1.18 | 1.33 | .185 | 0.16 |
| Age | 32.18 | 6.37 | 24.90 | 4.62 | -9.81 | <.001 | -1.21 |
| Politics | 3.58 | 1.09 | 3.28 | 1.07 | -1.90 | .058 | -0.23 |
| Favorability | 4.99 | 1.05 | 5.22 | 0.87 | 1.71 | .088 | 0.21 |
| Open | 4.87 | 1.41 | 5.19 | 1.10 | 1.85 | .065 | 0.23 |
| Conscientious | 4.85 | 1.16 | 4.93 | 1.13 | 0.48 | .632 | 0.06 |
| Extraverted | 4.72 | 1.37 | 5.02 | 1.23 | 1.62 | .107 | 0.20 |
| Agreeable | 5.22 | 1.20 | 5.40 | 1.13 | 1.60 | .110 | 0.20 |
| Neurotic | 3.35 | 1.62 | 3.28 | 1.45 | -0.32 | .749 | -0.04 |

# Supplementary Materials 3: Factor Loadings for Attribute Items (Experiment 2)

**Table S2**: *Rotated Factor Matrix for Attribute Items*

| Attribute Item | Factor Loading | |
| --- | --- | --- |
|  | 1 | 2 |
| Factor 1 – Warmth (*α* = .90) |  |  |
| 1. Likeable | **.90** | -.07 |
| 2. Warm | **.90** | -.09 |
| 3. Kind | **.88** | -.18 |
| 4. Agreeable | **.85** | -.20 |
| 5. Open | **.53** | .14 |
| 6. Conscientious | **.52** | .09 |
| Factor 2 – Competence (*α* = .75) |  |  |
| 7. Successful | .07 | **.74** |
| 8. Self-esteem | -.26 | **.73** |
| 9. Competent | .04 | **.68** |
| 10. Extraverted | -.02 | **.49** |

*Note*. Factor loadings > .40 are in boldface. Bartlett’s test of sphericity, *x^2^*(45) = 2435.08, *p* <.001; Kaiser-Meyer-Olkin = .86).

# Supplementary Materials 4: Alternative Mediation Models (Experiment 2)

## **Table S3**

*Summary of Alternative Mediation Analyses Where Mediator (Perceived Similarity) and Outcome Variables are Reversed*

| Mediator | Direct effect | Total effect | Indirect effect | |
| --- | --- | --- | --- | --- |
|  |  |  | Effect (Boot*SE*) | BS 95% CI |
| Warmth | 0.18 (0.08)* | 0.25 (0.09)** | -0.06 (0.05) | [-0.02, 0.16] |
| Competence | 0.25 (0.08)** | 0.25 (0.08)** | -0.01 (0.03) | [-0.06, 0.04] |
| Values | 0.22 (0.09)* | 0.25 (0.09)** | 0.03 (0.03) | [-0.01, 0.09] |
| Morality | 0.22 (0.07)** | 0.25 (0.08)** | 0.03 (0.04) | [-0.05, 0.12] |
| Altruistic job | 0.24 (0.08)** | 0.25 (0.09)** | 0.01 (0.01) | [-0.01, 0.04] |
| Collegiality | 0.16 (0.07)* | 0.25 (0.09)** | 0.09 (0.06) | [-0.02, 0.20] |
| Behavioral Intentions | 0.14 (0.07)* | 0.25 (0.08)** | 0.11 (0.06) | [-0.01, 0.23] |

*Note*. * *p* < .05, ** *p* < .01.The absence of any significant indirect effects suggests that the effect of rater narcissism on perceived similarity with the vanity narcissist is *not* mediated via perceived warmth, competence, values, morality, altruistic job suitability, collegiality, or behavioral intentions. These findings therefore support our proposed causal pathway: Rater Narcissism > Perceived Similarity > Outcome Variables. *N* = 215.

# Supplementary Materials 5: Mixed ANOVA Results (Experiment 2)

## **Table S4**

*Summary of 2 (Face type: Narcissist, Non-Narcissist) x 2 (Definition: Vanity, Selfishness) Mixed ANOVA Results*

| Outcome | Predictor | *F* | *η_p_*^2^ | *p* |
| --- | --- | --- | --- | --- |
| Attributes |  |  |  |  |
| Narcissistic | Face | 418.90 | .505 | <.001 |
|  | Definition | 4.49 | .011 | .035 |
|  | Face x Definition | 30.45 | .069 | <.001 |
| Selfish | Face | 567.00 | .580 | <.001 |
|  | Definition | 6.23 | .015 | .013 |
|  | Face x Definition | 12.90 | .031 | <.001 |
| Vain | Face | 232.15 | .362 | <.001 |
|  | Definition | 1.13 | .003 | .289 |
|  | Face x Definition | 130.97 | .243 | <.001 |
| Masculine | Face | 345.99 | .458 | <.001 |
|  | Definition | 8.72 | .021 | .003 |
|  | Face x Definition | 193.36 | .320 | <.001 |
| Age | Face | 2.88 | .007 | .091 |
|  | Definition | 1.97 | .005 | .161 |
|  | Face x Definition | 16.86 | .040 | <.001 |
| Politics | Face | 106.10 | .206 | <.001 |
|  | Definition | 2.08 | .005 | .150 |
|  | Face x Definition | 3.45 | .008 | .064 |
| Self-esteem | Face | 16.38 | .038 | <.001 |
|  | Definition | 11.42 | .027 | <.001 |
|  | Face x Definition | 185.04 | .311 | <.001 |
| Kind | Face | 772.73 | .653 | <.001 |
|  | Definition | 29.57 | .067 | <.001 |
|  | Face x Definition | 2.09 | .005 | .149 |
| Warm | Face | 834.35 | .671 | <.001 |
|  | Definition | 59.71 | .127 | <.001 |
|  | Face x Definition | 14.57 | .034 | <.001 |
| Likeable | Face | 541.54 | .569 | <.001 |
|  | Definition | 22.91 | .053 | <.001 |
|  | Face x Definition | 3.29 | .008 | .071 |
| Competent | Face | 0.43 | .001 | .512 |
|  | Definition | 2.13 | .005 | .146 |
|  | Face x Definition | 36.04 | .081 | <.001 |
| Successful | Face | 5.14 | .012 | .024 |
|  | Definition | 9.16 | .022 | .003 |
|  | Face x Definition | 125.89 | .235 | <.001 |
| Open | Face | 362.15 | .443 | <.001 |
|  | Definition | 51.92 | .112 | <.001 |
|  | Face x Definition | 74.83 | .154 | <.001 |
| Conscientious | Face | 99.95 | .196 | <.001 |
|  | Definition | 2.57 | .006 | .109 |
|  | Face x Definition | 1.80 | .004 | .181 |
| Extraverted | Face | 47.08 | .103 | <.001 |
|  | Definition | 46.65 | .102 | <.001 |
|  | Face x Definition | 231.90 | .361 | <.001 |
| Agreeable | Face | 696.25 | .629 | <.001 |
|  | Definition | 27.54 | .063 | <.001 |
|  | Face x Definition | 2.94 | .007 | .087 |
| Neurotic | Face | 11.94 | .028 | <.001 |
|  | Definition | 4.56 | .011 | .033 |
|  | Face x Definition | 0.84 | .002 | .361 |
| Values |  |  |  |  |
| Self-Transcendence | Face | 482.45 | .541 | <.001 |
|  | Definition | 17.63 | .041 | <.001 |
|  | Face x Definition | 1.59 | .004 | .208 |
| Self-Enhancement | Face | 229.94 | .359 | <.001 |
|  | Definition | 0.00 | .000 | .991 |
|  | Face x Definition | 33.88 | .076 | <.001 |
| Openness | Face | 114.19 | .218 | <.001 |
|  | Definition | 18.87 | .044 | <.001 |
|  | Face x Definition | 39.64 | .088 | <.001 |
| Conservation | Face | 125.15 | .234 | <.001 |
|  | Definition | 0.01 | .000 | .910 |
|  | Face x Definition | 2.95 | .007 | .087 |
| Moral Behaviours | Face | 666.29 | .619 | <.001 |
|  | Definition | 12.95 | .031 | <.001 |
|  | Face x Definition | 1.11 | .003 | .294 |
| Workplace |  |  |  |  |
| Corporate Management | Face | 17.08 | .040 | <.001 |
|  | Definition | 0.09 | .000 | .765 |
|  | Face x Definition | 38.94 | .087 | <.001 |
| Health Services | Face | 387.63 | .486 | <.001 |
|  | Definition | 23.32 | .054 | <.001 |
|  | Face x Definition | 8.08 | .019 | .005 |
| Boss | Face | 249.98 | .378 | <.001 |
|  | Definition | 31.83 | .072 | <.001 |
|  | Face x Definition | 6.90 | .017 | .009 |
| Colleague | Face | 307.52 | .429 | <.001 |
|  | Definition | 21.78 | .050 | <.001 |
|  | Face x Definition | 0.08 | .000 | .775 |
| Behavioural Outcomes |  |  |  |  |
| Prime Minister | Face | 103.97 | .202 | <.001 |
|  | Definition | 24.88 | .057 | <.001 |
|  | Face x Definition | 12.38 | .029 | <.001 |
| Trust | Face | 367.40 | .473 | <.001 |
|  | Definition | 14.62 | .034 | <.001 |
|  | Face x Definition | 4.88 | .012 | .028 |
| Lift | Face | 331.07 | .447 | <.001 |
|  | Definition | 13.48 | .032 | <.001 |
|  | Face x Definition | 0.67 | .002 | .414 |
| Similarity | Face | 138.14 | .252 | <.001 |
|  | Definition | 13.99 | .033 | <.001 |
|  | Face x Definition | 8.43 | .020 | .004 |

*Note. N* = 412.

# Supplementary Materials 6: Sample Demographics (Experiment 3)

## **Table S5**

*Sample Demographics*

|  | *N* |
| --- | --- |
| **Sex** |  |
| Male | 99 |
| Female | 101 |
| Prefer not to say | 2 |
| **Gender Identity** |  |
| Male | 99 |
| Female | 98 |
| Trans Man | 1 |
| Trans Woman | 1 |
| Non-Binary | 1 |
| Prefer not to say | 2 |
| **Sexual Orientation** |  |
| Heterosexual | 174 |
| Gay | 8 |
| Lesbian | 3 |
| Bisexual | 11 |
| Prefer not to say | 4 |
| Other | 2 |
| **Asexual** |  |
| Yes | 3 |
| No | 195 |
| Prefer not to say | 4 |
| **Relationship Status** |  |
| In a relationship | 139 |
| Not in a relationship | 59 |
| Prefer not to say | 4 |

# Supplementary Materials 7: Associations Between Additional Experiment Measures and Evaluations of the Four Faces (Experiment 3)

In Experiment 3, in addition to the Single Items Narcissism Scale (SINS; Konrath et al., 2014), participants also completed the following measures :the Narcissistic Personality Inventory (NPI-13; Gentile et al., 2013), the Narcissistic Admiration and Rivalry Questionnaire Short Version (NARQ-S; Leckelt et al., 2018), a shortened version of the Attraction to Narcissistic Personality measure (ANP; Haslam & Montrose, 2015), the Emotional Promiscuity scale (EP; Jones, 2011), the Experience in Close Relationships Short Form (ECR_SF; Wei et al., 2007). Tables 25-44 Show associations between participants scores on these measures and their evaluations of the four faces (selfishness narcissist, non-selfishness narcissist, vanity narcissist, non-vanity narcissist).

## **Table S6**

*Bonferroni-corrected Spearman’s correlations for NPI-13 Score and Evaluations of the Selfishness Narcissist*

|  | 1 | 2 | 3 | 4 | 5 | 6 | 7 | 8 | 9 | 10 |
| --- | --- | --- | --- | --- | --- | --- | --- | --- | --- | --- |
| 1. NPI |  |  |  |  |  |  |  |  |  |  |
| 2. Romantic Suitability | **-0.01** |  |  |  |  |  |  |  |  |  |
| 3. Toxic Behaviours | **0.09** | -0.22** |  |  |  |  |  |  |  |  |
| 4. Familiarity | **0.08** | 0.37** | -0.10 |  |  |  |  |  |  |  |
| 5. Similarity | **0.01** | 0.42** | -0.41** | 0.35** |  |  |  |  |  |  |
| 6. Warmth | **0.12** | 0.46** | -0.33** | 0.26** | 0.46** |  |  |  |  |  |
| 7. Competence | **-0.11** | 0.52** | -0.37** | 0.30** | 0.39** | 0.28** |  |  |  |  |
| 8. Masculinity | **-0.20**+** | 0.14 | 0.05 | 0.03 | -0.10 | -0.10 | 0.12 |  |  |  |
| 9. Secret Enjoyment | **0.01** | 0.48** | -0.11 | 0.38** | 0.38** | 0.35** | 0.26** | 0.04 |  |  |
| 10. Narcissism | **0.05** | -0.18** | 0.42** | -0.06 | -0.30** | -0.32** | -0.27** | 0.03 | -0.08 |  |

*Note.* * *p* < .05; ** *p* < .01; + *p* < adjusted *α* = 0.006 (0.05/9). ‘Romantic Suitability’ is an index of participants’ average scores on perceived attraction, suitability for short- and long-term partnership (general and personal), and suitability for friendship (*α* = .84). *N* = 202.

## **Table S7**

*Bonferroni-corrected Spearman’s correlations for NPI-13 Score and Evaluations of the Non-Selfishness Narcissist*

|  | 1 | 2 | 3 | 4 | 5 | 6 | 7 | 8 | 9 | 10 |
| --- | --- | --- | --- | --- | --- | --- | --- | --- | --- | --- |
| 1. NPI |  |  |  |  |  |  |  |  |  |  |
| 2. Romantic Suitability | **0.03** |  |  |  |  |  |  |  |  |  |
| 3. Toxic Behaviours | **0.11** | -0.45** |  |  |  |  |  |  |  |  |
| 4. Familiarity | **0.04** | 0.35** | -0.01 |  |  |  |  |  |  |  |
| 5. Similarity | **-0.18*** | 0.55** | -0.35** | 0.30** |  |  |  |  |  |  |
| 6. Warmth | **-0.03** | 0.70** | -0.40** | 0.22** | 0.53** |  |  |  |  |  |
| 7. Competence | **-0.10** | 0.49** | -0.28** | 0.11 | 0.44** | 0.47** |  |  |  |  |
| 8. Masculinity | **-0.00** | -0.10 | 0.24** | 0.05 | -0.03 | -0.10 | 0.03 |  |  |  |
| 9. Secret Enjoyment | **-0.01** | 0.58** | -0.28** | 0.41** | 0.47** | 0.46** | 0.30** | -0.02 |  |  |
| 10. Narcissism | **0.03** | -0.20** | 0.24** | -0.09 | -0.18* | -0.30** | -0.12 | 0.13 | -0.12 |  |

*Note.* * *p* < .05; ** *p* < .01; + *p* < adjusted *α* = 0.006 (0.05/9). ‘Romantic Suitability’ is an index of participants’ average scores on perceived attraction, suitability for short- and long-term partnership (general and personal), and suitability for friendship (*α* = .84). *N* = 202.

## **Table S8**

*Bonferroni-corrected Spearman’s correlations for NPI-13 Score and Evaluations of the Vanity Narcissist*

|  | 1 | 2 | 3 | 4 | 5 | 6 | 7 | 8 | 9 | 10 |
| --- | --- | --- | --- | --- | --- | --- | --- | --- | --- | --- |
| 1. NPI |  |  |  |  |  |  |  |  |  |  |
| 2. Romantic Suitability | **0.07** |  |  |  |  |  |  |  |  |  |
| 3. Toxic Behaviours | **0.02** | -0.16* |  |  |  |  |  |  |  |  |
| 4. Familiarity | **0.16*** | 0.49** | -0.11 |  |  |  |  |  |  |  |
| 5. Similarity | **0.04** | 0.36** | -0.36** | 0.29** |  |  |  |  |  |  |
| 6. Warmth | **0.07** | 0.46** | -0.34** | 0.30** | 0.40** |  |  |  |  |  |
| 7. Competence | **-0.06** | 0.44** | -0.18* | 0.20** | 0.20** | 0.19** |  |  |  |  |
| 8. Masculinity | **-0.05** | -0.13 | 0.17* | -0.05 | -0.09 | -0.16* | 0.05 |  |  |  |
| 9. Secret Enjoyment | **0.11** | 0.53** | -0.13 | 0.44** | 0.28** | 0.26** | 0.27** | -0.09 |  |  |
| 10. Narcissism | **0.11** | -0.08 | 0.49** | -0.15* | -0.42** | -0.29** | -0.01 | 0.12 | 0.02 |  |

*Note.* * *p* < .05; ** *p* < .01; + *p* < adjusted *α* = 0.006 (0.05/9). ‘Romantic Suitability’ is an index of participants’ average scores on perceived attraction, suitability for short- and long-term partnership (general and personal), and suitability for friendship (*α* = .84). *N* = 202.

## **Table S9**

*Bonferroni-corrected Spearman’s correlations for NPI-13 Score and Evaluations of the Non-Vanity Narcissist*

|  | 1 | 2 | 3 | 4 | 5 | 6 | 7 | 8 | 9 | 10 |
| --- | --- | --- | --- | --- | --- | --- | --- | --- | --- | --- |
| 1. NPI |  |  |  |  |  |  |  |  |  |  |
| 2. Romantic Suitability | **-0.04** |  |  |  |  |  |  |  |  |  |
| 3. Toxic Behaviours | **0.07** | -0.22** |  |  |  |  |  |  |  |  |
| 4. Familiarity | **-0.00** | 0.33** | -0.07 |  |  |  |  |  |  |  |
| 5. Similarity | **-0.11** | 0.41** | -0.23** | 0.29** |  |  |  |  |  |  |
| 6. Warmth | **-0.04** | 0.52** | -0.33** | 0.26** | 0.37** |  |  |  |  |  |
| 7. Competence | **-0.11** | 0.46** | -0.08 | 0.19** | 0.23** | 0.31** |  |  |  |  |
| 8. Masculinity | **-0.15*** | 0.22** | 0.09 | 0.04 | -0.00 | 0.08 | 0.23** |  |  |  |
| 9. Secret Enjoyment | **-0.02** | 0.53** | -0.23** | 0.32** | 0.31** | 0.30** | 0.35** | 0.00 |  |  |
| 10. Narcissism | **0.04** | -0.01 | 0.42** | -0.06 | -0.09 | -0.22** | 0.03 | 0.09 | 0.07 |  |

*Note.* * *p* < .05; ** *p* < .01; + *p* < adjusted *α* = 0.006 (0.05/9). ‘Romantic Suitability’ is an index of participants’ average scores on perceived attraction, suitability for short- and long-term partnership (general and personal), and suitability for friendship (*α* = .84). *N* = 202

## **Table S10**

*Bonferroni-corrected Spearman’s correlations for NARQ Score and Evaluations of the Selfishness Narcissist*

|  | 1 | 2 | 3 | 4 | 5 | 6 | 7 | 8 | 9 | 10 |
| --- | --- | --- | --- | --- | --- | --- | --- | --- | --- | --- |
| 1. NPI |  |  |  |  |  |  |  |  |  |  |
| 2. Romantic Suitability | **-0.02** |  |  |  |  |  |  |  |  |  |
| 3. Toxic Behaviours | **0.11** | -0.22** |  |  |  |  |  |  |  |  |
| 4. Familiarity | **0.12** | 0.37** | -0.10 |  |  |  |  |  |  |  |
| 5. Similarity | **-0.03** | 0.42** | -0.41** | 0.35** |  |  |  |  |  |  |
| 6. Warmth | **0.02** | 0.46** | -0.33** | 0.26** | 0.46** |  |  |  |  |  |
| 7. Competence | **-0.12** | 0.52** | -0.37** | 0.30** | 0.39** | 0.28** |  |  |  |  |
| 8. Masculinity | **-0.14** | 0.14 | 0.05 | 0.03 | -0.10 | -0.10 | 0.12 |  |  |  |
| 9. Secret Enjoyment | **0.07** | 0.48** | -0.11 | 0.38** | 0.38** | 0.35** | 0.26** | 0.04 |  |  |
| 10. Narcissism | **0.15*** | -0.18** | 0.42** | -0.06 | -0.30** | -0.32** | -0.27** | 0.03 | -0.08 |  |

*Note.* * *p* < .05; ** *p* < .01; + *p* < adjusted *α* = 0.006 (0.05/9). Romantic Suitability’ is an index of participants’ average scores on perceived attraction, suitability for short- and long-term partnership (general and personal), and suitability for friendship (*α* = .84). *N* = 202.

## **Table S11**

*Bonferroni-corrected Spearman’s correlations for NARQ Score and Evaluations of the Non-Selfishness Narcissist*

|  | 1 | 2 | 3 | 4 | 5 | 6 | 7 | 8 | 9 | 10 |
| --- | --- | --- | --- | --- | --- | --- | --- | --- | --- | --- |
| 1. NPI |  |  |  |  |  |  |  |  |  |  |
| 2. Romantic Suitability | **0.04** |  |  |  |  |  |  |  |  |  |
| 3. Toxic Behaviours | **0.08** | -0.45** |  |  |  |  |  |  |  |  |
| 4. Familiarity | **0.07** | 0.35** | -0.01 |  |  |  |  |  |  |  |
| 5. Similarity | **-0.11** | 0.55** | -0.35** | 0.30** |  |  |  |  |  |  |
| 6. Warmth | **-0.07** | 0.70** | -0.40** | 0.22** | 0.53** |  |  |  |  |  |
| 7. Competence | **-0.22**+** | 0.49** | -0.28** | 0.11 | 0.44** | 0.47** |  |  |  |  |
| 8. Masculinity | **-0.02** | -0.10 | 0.24** | 0.05 | -0.03 | -0.10 | 0.03 |  |  |  |
| 9. Secret Enjoyment | **0.11** | 0.58** | -0.28** | 0.41** | 0.47** | 0.46** | 0.30** | -0.02 |  |  |
| 10. Narcissism | **0.01** | -0.20** | 0.24** | -0.09 | -0.18* | -0.30** | -0.12 | 0.13 | -0.12 |  |

*Note.* * *p* < .05; ** *p* < .01; + *p* < adjusted *α* = 0.006 (0.05/9). ‘Romantic Suitability’ is an index of participants’ average scores on perceived attraction, suitability for short- and long-term partnership (general and personal), and suitability for friendship (*α* = .84). *N* = 202.

## **Table S12**

*Bonferroni-corrected Spearman’s correlations for NARQ Score and Evaluations of the Vanity Narcissist*

|  | 1 | 2 | 3 | 4 | 5 | 6 | 7 | 8 | 9 | 10 |
| --- | --- | --- | --- | --- | --- | --- | --- | --- | --- | --- |
| 1. NPI |  |  |  |  |  |  |  |  |  |  |
| 2. Romantic Suitability | **0.04** |  |  |  |  |  |  |  |  |  |
| 3. Toxic Behaviours | **-0.01** | -0.16* |  |  |  |  |  |  |  |  |
| 4. Familiarity | **0.12** | 0.49** | -0.11 |  |  |  |  |  |  |  |
| 5. Similarity | **0.01** | 0.36** | -0.36** | 0.29** |  |  |  |  |  |  |
| 6. Warmth | **0.01** | 0.46** | -0.34** | 0.30** | 0.40** |  |  |  |  |  |
| 7. Competence | **-0.07** | 0.44** | -0.18* | 0.20** | 0.20** | 0.19** |  |  |  |  |
| 8. Masculinity | **-0.06** | -0.13 | 0.17* | -0.05 | -0.09 | -0.16* | 0.05 |  |  |  |
| 9. Secret Enjoyment | **0.18**** | 0.53** | -0.13* | 0.44** | 0.28** | 0.26** | 0.27** | -0.09 |  |  |
| 10. Narcissism | **0.20**+** | -0.08 | 0.49** | -0.15* | -0.42** | -0.29** | -0.01 | 0.12 | 0.02 |  |

*Note.* * *p* < .05; ** *p* < .01; + *p* < adjusted *α* = 0.006 (0.05/9). Romantic Suitability’ is an index of participants’ average scores on perceived attraction, suitability for short- and long-term partnership (general and personal), and suitability for friendship (*α* = .84). *N* = 202.

## **Table S13**

*Bonferroni-corrected Spearman’s correlations for NARQ Score and Evaluations of the Non-Vanity Narcissist*

|  | 1 | 2 | 3 | 4 | 5 | 6 | 7 | 8 | 9 | 10 |
| --- | --- | --- | --- | --- | --- | --- | --- | --- | --- | --- |
| 1. NPI |  |  |  |  |  |  |  |  |  |  |
| 2. Romantic Suitability | **-0.04** |  |  |  |  |  |  |  |  |  |
| 3. Toxic Behaviours | **0.06** | -0.22** |  |  |  |  |  |  |  |  |
| 4. Familiarity | **0.01** | 0.33** | -0.07 |  |  |  |  |  |  |  |
| 5. Similarity | **-0.13** | 0.41** | -0.23** | 0.29** |  |  |  |  |  |  |
| 6. Warmth | **-0.06** | 0.52** | -0.33** | 0.26** | 0.37** |  |  |  |  |  |
| 7. Competence | **-0.11** | 0.46** | -0.08 | 0.19** | 0.23** | 0.31** |  |  |  |  |
| 8. Masculinity | **-0.16*** | 0.22** | 0.09 | 0.04 | -0.00 | 0.08 | 0.23** |  |  |  |
| 9. Secret Enjoyment | **0.06** | 0.53** | -0.23** | 0.32** | 0.31** | 0.30** | 0.35** | 0.00 |  |  |
| 10. Narcissism | **0.04** | -0.01 | 0.42** | -0.06 | -0.09 | -0.22** | 0.03 | 0.09 | 0.07 |  |

*Note.* * *p* < .05; ** *p* < .01; + *p* < adjusted *α* = 0.006 (0.05/9). ‘Romantic Suitability’ is an index of participants’ average scores on perceived attraction, suitability for short- and long-term partnership (general and personal), and suitability for friendship (*α* = .84). *N* = 202.

## **Table S14**

*Bonferroni-corrected Spearman’s correlations for Attraction to Narcissistic Personality (ANP) Score and Evaluations of the Selfishness Narcissist*

|  | 1 | 2 | 3 | 4 | 5 | 6 | 7 | 8 | 9 | 10 |
| --- | --- | --- | --- | --- | --- | --- | --- | --- | --- | --- |
| 1. NPI |  |  |  |  |  |  |  |  |  |  |
| 2. Romantic Suitability | **0.02** |  |  |  |  |  |  |  |  |  |
| 3. Toxic Behaviours | **-0.06** | -0.22** |  |  |  |  |  |  |  |  |
| 4. Familiarity | **0.03** | 0.37** | -0.10 |  |  |  |  |  |  |  |
| 5. Similarity | **0.01** | 0.42** | -0.41** | 0.35** |  |  |  |  |  |  |
| 6. Warmth | **0.03** | 0.46** | -0.33** | 0.26** | 0.46** |  |  |  |  |  |
| 7. Competence | **0.01** | 0.52** | -0.37** | 0.30** | 0.39** | 0.28** |  |  |  |  |
| 8. Masculinity | **-0.00** | 0.14 | 0.05 | 0.03 | -0.10 | -0.10 | 0.12 |  |  |  |
| 9. Secret Enjoyment | **0.01** | 0.48** | -0.11 | 0.38** | 0.38** | 0.35** | 0.26** | 0.04 |  |  |
| 10. Narcissism | **0.03** | -0.18** | 0.42** | -0.06 | -0.30** | -0.32** | -0.27** | 0.03 | -0.08 |  |

*Note.* * *p* < .05; ** *p* < .01; + *p* < adjusted *α* = 0.006 (0.05/9). ‘Romantic Suitability’ is an index of participants’ average scores on perceived attraction, suitability for short- and long-term partnership (general and personal), and suitability for friendship (*α* = .84). *N* = 202.

## **Table S15**

*Bonferroni-corrected Spearman’s correlations for Attraction to Narcissistic Personality (ANP) Score and Evaluations of the Non-Selfishness Narcissist*

|  | 1 | 2 | 3 | 4 | 5 | 6 | 7 | 8 | 9 | 10 |
| --- | --- | --- | --- | --- | --- | --- | --- | --- | --- | --- |
| 1. NPI |  |  |  |  |  |  |  |  |  |  |
| 2. Romantic Suitability | **0.05** |  |  |  |  |  |  |  |  |  |
| 3. Toxic Behaviours | **0.14*** | -0.45** |  |  |  |  |  |  |  |  |
| 4. Familiarity | **0.08** | 0.35** | -0.01 |  |  |  |  |  |  |  |
| 5. Similarity | **-0.11** | 0.55** | -0.35** | 0.30** |  |  |  |  |  |  |
| 6. Warmth | **-0.04** | 0.70** | -0.40** | 0.22** | 0.53** |  |  |  |  |  |
| 7. Competence | **-0.06** | 0.49** | -0.28** | 0.11 | 0.44** | 0.47** |  |  |  |  |
| 8. Masculinity | **-0.03** | -0.10 | 0.24** | 0.05 | -0.03 | -0.10 | 0.03 |  |  |  |
| 9. Secret Enjoyment | **0.07** | 0.58** | -0.28** | 0.41** | 0.47** | 0.46** | 0.30** | -0.02 |  |  |
| 10. Narcissism | **0.11** | -0.20** | 0.24** | -0.09 | -0.18* | -0.30** | -0.12 | 0.13 | -0.12 |  |

*Note.* * *p* < .05; ** *p* < .01; + *p* < adjusted *α* = 0.006 (0.05/9). ‘Romantic Suitability’ is an index of participants’ average scores on perceived attraction, suitability for short- and long-term partnership (general and personal), and suitability for friendship (*α* = .84). *N* = 202.

## **Table S16**

*Bonferroni-corrected Spearman’s correlations for Attraction to Narcissistic Personality (ANP) Score and Evaluations of the Vanity Narcissist*

|  | 1 | 2 | 3 | 4 | 5 | 6 | 7 | 8 | 9 | 10 |
| --- | --- | --- | --- | --- | --- | --- | --- | --- | --- | --- |
| 1. NPI |  |  |  |  |  |  |  |  |  |  |
| 2. Romantic Suitability | **0.01** |  |  |  |  |  |  |  |  |  |
| 3. Toxic Behaviours | **-0.05** | -0.16* |  |  |  |  |  |  |  |  |
| 4. Familiarity | **0.11** | 0.49** | -0.11 |  |  |  |  |  |  |  |
| 5. Similarity | **-0.04** | 0.36** | -0.36** | 0.29** |  |  |  |  |  |  |
| 6. Warmth | **-0.05** | 0.46** | -0.34** | 0.30** | 0.40** |  |  |  |  |  |
| 7. Competence | **0.04** | 0.44** | -0.18* | 0.20** | 0.20** | 0.19** |  |  |  |  |
| 8. Masculinity | **0.07** | -0.13 | 0.17* | -0.05 | -0.09 | -0.16* | 0.05 |  |  |  |
| 9. Secret Enjoyment | **0.12** | 0.53** | -0.13 | 0.44** | 0.28** | 0.26** | 0.27** | -0.09 |  |  |
| 10. Narcissism | **0.10** | -0.08 | 0.49** | -0.15* | -0.42** | -0.29** | -0.01 | 0.12 | 0.02 |  |

*Note.* * *p* < .05; ** *p* < .01; + *p* < adjusted *α* = 0.006 (0.05/9). ‘Romantic Suitability’ is an index of participants’ average scores on perceived attraction, suitability for short- and long-term partnership (general and personal), and suitability for friendship (*α* = .84). *N* = 202.

## **Table S17**

*Bonferroni-corrected Spearman’s correlations for Attraction to Narcissistic Personality (ANP) Score and Evaluations of the Non-Vanity Narcissist*

|  | 1 | 2 | 3 | 4 | 5 | 6 | 7 | 8 | 9 | 10 |
| --- | --- | --- | --- | --- | --- | --- | --- | --- | --- | --- |
| 1. NPI |  |  |  |  |  |  |  |  |  |  |
| 2. Romantic Suitability | **0.02** |  |  |  |  |  |  |  |  |  |
| 3. Toxic Behaviours | **-0.03** | -0.22** |  |  |  |  |  |  |  |  |
| 4. Familiarity | **0.01** | 0.33** | -0.07 |  |  |  |  |  |  |  |
| 5. Similarity | **-0.08** | 0.41** | -0.23** | 0.29** |  |  |  |  |  |  |
| 6. Warmth | **-0.02** | 0.52** | -0.33** | 0.26** | 0.37** |  |  |  |  |  |
| 7. Competence | **-0.10** | 0.46** | -0.08 | 0.19** | 0.23** | 0.31** |  |  |  |  |
| 8. Masculinity | **-0.10** | 0.22** | 0.09 | 0.04 | -0.00 | 0.08 | 0.23** |  |  |  |
| 9. Secret Enjoyment | **0.07** | 0.53** | -0.23** | 0.32** | 0.31** | 0.30** | 0.35** | 0.00 |  |  |
| 10. Narcissism | **0.01** | -0.01 | 0.42** | -0.06 | -0.09 | -0.22** | 0.03 | 0.09 | 0.07 |  |

*Note.* * *p* < .05; ** *p* < .01; + *p* < adjusted *α* = 0.006 (0.05/9). ‘Romantic Suitability’ is an index of participants’ average scores on perceived attraction, suitability for short- and long-term partnership (general and personal), and suitability for friendship (*α* = .84). *N* = 202.

## **Table S18**

*Bonferroni-corrected Spearman’s correlations for Emotional Promiscuity (EP) Score and Evaluations of the Selfishness Narcissist*

|  | 1 | 2 | 3 | 4 | 5 | 6 | 7 | 8 | 9 | 10 |
| --- | --- | --- | --- | --- | --- | --- | --- | --- | --- | --- |
| 1. NPI |  |  |  |  |  |  |  |  |  |  |
| 2. Romantic Suitability | **-0.06** |  |  |  |  |  |  |  |  |  |
| 3. Toxic Behaviours | **0.02** | -0.22** |  |  |  |  |  |  |  |  |
| 4. Familiarity | **0.11** | 0.37** | -0.10 |  |  |  |  |  |  |  |
| 5. Similarity | **0.01** | 0.42** | -0.41** | 0.35** |  |  |  |  |  |  |
| 6. Warmth | **0.03** | 0.46** | -0.33** | 0.26** | 0.46** |  |  |  |  |  |
| 7. Competence | **-0.09** | 0.52** | -0.37** | 0.30** | 0.39** | 0.28** |  |  |  |  |
| 8. Masculinity | **-0.18**** | 0.14 | 0.05 | 0.03 | -0.10 | -0.10 | 0.12 |  |  |  |
| 9. Secret Enjoyment | **0.06** | 0.48** | -0.11 | 0.38** | 0.38** | 0.35** | 0.26** | 0.04 |  |  |
| 10. Narcissism | **0.00** | -0.18** | 0.42** | -0.06 | -0.30** | -0.32** | -0.27** | 0.03 | -0.08 |  |

*Note.* * *p* < .05; ** *p* < .01; + *p* < adjusted *α* = 0.006 (0.05/9). ‘Romantic Suitability’ is an index of participants’ average scores on perceived attraction, suitability for short- and long-term partnership (general and personal), and suitability for friendship (*α* = .84). *N* = 202.

## **Table S19**

*Bonferroni-corrected Spearman’s correlations for Emotional Promiscuity (EP) Score and Evaluations of the Non-Selfishness Narcissist*

|  | 1 | 2 | 3 | 4 | 5 | 6 | 7 | 8 | 9 | 10 |
| --- | --- | --- | --- | --- | --- | --- | --- | --- | --- | --- |
| 1. NPI |  |  |  |  |  |  |  |  |  |  |
| 2. Romantic Suitability | **0.07** |  |  |  |  |  |  |  |  |  |
| 3. Toxic Behaviours | **-0.02** | -0.45** |  |  |  |  |  |  |  |  |
| 4. Familiarity | **0.09** | 0.35** | -0.01 |  |  |  |  |  |  |  |
| 5. Similarity | **0.00** | 0.55** | -0.35** | 0.30** |  |  |  |  |  |  |
| 6. Warmth | **-0.08** | 0.70** | -0.40** | 0.22** | 0.53** |  |  |  |  |  |
| 7. Competence | **-0.16*** | 0.49** | -0.28** | 0.11 | 0.44** | 0.47** |  |  |  |  |
| 8. Masculinity | **0.03** | -0.10 | 0.24** | 0.05 | -0.03 | -0.10 | 0.03 |  |  |  |
| 9. Secret Enjoyment | **0.03** | 0.58** | -0.28** | 0.41** | 0.47** | 0.46** | 0.30** | -0.02 |  |  |
| 10. Narcissism | **0.09** | -0.20** | 0.24** | -0.09 | -0.18* | -0.30** | -0.12 | 0.13 | -0.12 |  |

*Note.* * *p* < .05; ** *p* < .01; + *p* < adjusted *α* = 0.006 (0.05/9). ‘Romantic Suitability’ is an index of participants’ average scores on perceived attraction, suitability for short- and long-term partnership (general and personal), and suitability for friendship (*α* = .84). *N* = 202.

## **Table S20**

*Bonferroni-corrected Spearman’s correlations for Emotional Promiscuity (EP) Score and Evaluations of the Vanity Narcissist*

|  | 1 | 2 | 3 | 4 | 5 | 6 | 7 | 8 | 9 | 10 |
| --- | --- | --- | --- | --- | --- | --- | --- | --- | --- | --- |
| 1. NPI |  |  |  |  |  |  |  |  |  |  |
| 2. Romantic Suitability | **-0.01** |  |  |  |  |  |  |  |  |  |
| 3. Toxic Behaviours | **-0.09** | -0.16* |  |  |  |  |  |  |  |  |
| 4. Familiarity | **0.05** | 0.49** | -0.11 |  |  |  |  |  |  |  |
| 5. Similarity | **0.08** | 0.36** | -0.36** | 0.29** |  |  |  |  |  |  |
| 6. Warmth | **0.05** | 0.46** | -0.34** | 0.30** | 0.40** |  |  |  |  |  |
| 7. Competence | **-0.09** | 0.44** | -0.18* | 0.20** | 0.20** | 0.19** |  |  |  |  |
| 8. Masculinity | **-0.01** | -0.13 | 0.17* | -0.05 | -0.09 | -0.16* | 0.05 |  |  |  |
| 9. Secret Enjoyment | **0.04** | 0.53** | -0.13 | 0.44** | 0.28** | 0.26** | 0.27** | -0.09 |  |  |
| 10. Narcissism | **0.00** | -0.08 | 0.49** | -0.15* | -0.42** | -0.29** | -0.01 | 0.12 | 0.02 |  |

*Note.* * *p* < .05; ** *p* < .01; + *p* < adjusted *α* = 0.006 (0.05/9). ‘Romantic Suitability’ is an index of participants’ average scores on perceived attraction, suitability for short- and long-term partnership (general and personal), and suitability for friendship (*α* = .84). *N* = 202.

## **Table S21**

*Bonferroni-corrected Spearman’s correlations for Emotional Promiscuity (EP) Score and Evaluations of the Non-Vanity Narcissist*

|  | 1 | 2 | 3 | 4 | 5 | 6 | 7 | 8 | 9 | 10 |
| --- | --- | --- | --- | --- | --- | --- | --- | --- | --- | --- |
| 1. NPI |  |  |  |  |  |  |  |  |  |  |
| 2. Romantic Suitability | **-0.05** |  |  |  |  |  |  |  |  |  |
| 3. Toxic Behaviours | **0.01** | -0.22** |  |  |  |  |  |  |  |  |
| 4. Familiarity | **0.04** | 0.33** | -0.07 |  |  |  |  |  |  |  |
| 5. Similarity | **-0.05** | 0.41** | -0.23** | 0.29** |  |  |  |  |  |  |
| 6. Warmth | **-0.01** | 0.52** | -0.33** | 0.26** | 0.37** |  |  |  |  |  |
| 7. Competence | **-0.11** | 0.46** | -0.08 | 0.19** | 0.23** | 0.31** |  |  |  |  |
| 8. Masculinity | **-0.06** | 0.22** | 0.09 | 0.04 | -0.00 | 0.08 | 0.23** |  |  |  |
| 9. Secret Enjoyment | **0.04** | 0.53** | -0.23** | 0.32** | 0.31** | 0.30** | 0.35** | 0.00 |  |  |
| 10. Narcissism | **0.03** | -0.01 | 0.42** | -0.06 | -0.09 | -0.22** | 0.03 | 0.09 | 0.07 |  |

*Note.* * *p* < .05; ** *p* < .01; + *p* < adjusted *α* = 0.006 (0.05/9). ‘Romantic Suitability’ is an index of participants’ average scores on perceived attraction, suitability for short- and long-term partnership (general and personal), and suitability for friendship (*α* = .84). *N* = 202.

## **Table S22**

*Bonferroni-corrected Spearman’s correlations for ECR-S (Anxiety) Score and Evaluations of the Selfishness Narcissist*

|  | 1 | 2 | 3 | 4 | 5 | 6 | 7 | 8 | 9 | 10 |
| --- | --- | --- | --- | --- | --- | --- | --- | --- | --- | --- |
| 1. NPI |  |  |  |  |  |  |  |  |  |  |
| 2. Romantic Suitability | **0.08** |  |  |  |  |  |  |  |  |  |
| 3. Toxic Behaviours | **0.10** | -0.22** |  |  |  |  |  |  |  |  |
| 4. Familiarity | **0.15*** | 0.37** | -0.10 |  |  |  |  |  |  |  |
| 5. Similarity | **-0.02** | 0.42** | -0.41** | 0.35** |  |  |  |  |  |  |
| 6. Warmth | **-0.03** | 0.46** | -0.33** | 0.26** | 0.46** |  |  |  |  |  |
| 7. Competence | **0.03** | 0.52** | -0.37** | 0.30** | 0.39** | 0.28** |  |  |  |  |
| 8. Masculinity | **0.10** | 0.14 | 0.05 | 0.03 | -0.10 | -0.10 | 0.12 |  |  |  |
| 9. Secret Enjoyment | **0.07** | 0.48** | -0.11 | 0.38** | 0.38** | 0.35** | 0.26** | 0.04 |  |  |
| 10. Narcissism | **0.07** | -0.18** | 0.42** | -0.06 | -0.30** | -0.32** | -0.27** | 0.03 | -0.08 |  |

*Note.* * *p* < .05; ** *p* < .01; + *p* < adjusted *α* = 0.006 (0.05/9). ‘Romantic Suitability’ is an index of participants’ average scores on perceived attraction, suitability for short- and long-term partnership (general and personal), and suitability for friendship (*α* = .84). *N* = 202.

## **Table S23**

*Bonferroni-corrected Spearman’s correlations for ECR-S (Anxiety) Score and Evaluations of the Non-Selfishness Narcissist*

|  | 1 | 2 | 3 | 4 | 5 | 6 | 7 | 8 | 9 | 10 |
| --- | --- | --- | --- | --- | --- | --- | --- | --- | --- | --- |
| 1. NPI |  |  |  |  |  |  |  |  |  |  |
| 2. Romantic Suitability | **0.02** |  |  |  |  |  |  |  |  |  |
| 3. Toxic Behaviours | **-0.09** | -0.45** |  |  |  |  |  |  |  |  |
| 4. Familiarity | **0.08** | 0.35** | -0.01 |  |  |  |  |  |  |  |
| 5. Similarity | **0.02** | 0.55** | -0.35** | 0.30** |  |  |  |  |  |  |
| 6. Warmth | **0.04** | 0.70** | -0.40** | 0.22** | 0.53** |  |  |  |  |  |
| 7. Competence | **-0.11** | 0.49** | -0.28** | 0.11 | 0.44** | 0.47** |  |  |  |  |
| 8. Masculinity | **0.02** | -0.10 | 0.24** | 0.05 | -0.03 | -0.10 | 0.03 |  |  |  |
| 9. Secret Enjoyment | **0.12** | 0.58** | -0.28** | 0.41** | 0.47** | 0.46** | 0.30** | -0.02 |  |  |
| 10. Narcissism | **-0.01** | -0.20** | 0.24** | -0.09 | -0.18* | -0.30** | -0.12 | 0.13 | -0.12 |  |

*Note.* * *p* < .05; ** *p* < .01; + *p* < adjusted *α* = 0.006 (0.05/9). ‘Romantic Suitability’ is an index of participants’ average scores on perceived attraction, suitability for short- and long-term partnership (general and personal), and suitability for friendship (*α* = .84). *N* = 202.

## **Table S24**

*Bonferroni-corrected Spearman’s correlations for ECR-S (Anxiety) Score and Evaluations of the Vanity Narcissist*

|  | 1 | 2 | 3 | 4 | 5 | 6 | 7 | 8 | 9 | 10 |
| --- | --- | --- | --- | --- | --- | --- | --- | --- | --- | --- |
| 1. NPI |  |  |  |  |  |  |  |  |  |  |
| 2. Romantic Suitability | **-0.01** |  |  |  |  |  |  |  |  |  |
| 3. Toxic Behaviours | **-0.00** | -0.16* |  |  |  |  |  |  |  |  |
| 4. Familiarity | **0.12** | 0.49** | -0.11 |  |  |  |  |  |  |  |
| 5. Similarity | **-0.03** | 0.36** | -0.36** | 0.29** |  |  |  |  |  |  |
| 6. Warmth | **-0.04** | 0.46** | -0.34** | 0.30** | 0.40** |  |  |  |  |  |
| 7. Competence | **0.00** | 0.44** | -0.18* | 0.20** | 0.20** | 0.19** |  |  |  |  |
| 8. Masculinity | **0.12** | -0.13 | 0.17* | -0.05 | -0.09 | -0.16* | 0.05 |  |  |  |
| 9. Secret Enjoyment | **0.10** | 0.53** | -0.13 | 0.44** | 0.28** | 0.26** | 0.27** | -0.09 |  |  |
| 10. Narcissism | **0.17*** | -0.08 | 0.49** | -0.15* | -0.42** | -0.29** | -0.01 | 0.12 | 0.02 |  |

*Note.* * *p* < .05; ** *p* < .01; + *p* < adjusted *α* = 0.006 (0.05/9). ‘Romantic Suitability’ is an index of participants’ average scores on perceived attraction, suitability for short- and long-term partnership (general and personal), and suitability for friendship (*α* = .84). *N* = 202.

## **Table S25**

*Bonferroni-corrected Spearman’s correlations for ECR-S (Anxiety) Score and Evaluations of the Non-Vanity Narcissist*

|  | 1 | 2 | 3 | 4 | 5 | 6 | 7 | 8 | 9 | 10 |
| --- | --- | --- | --- | --- | --- | --- | --- | --- | --- | --- |
| 1. NPI |  |  |  |  |  |  |  |  |  |  |
| 2. Romantic Suitability | **0.05** |  |  |  |  |  |  |  |  |  |
| 3. Toxic Behaviours | **-0.08** | -0.22** |  |  |  |  |  |  |  |  |
| 4. Familiarity | **0.16*** | 0.33** | -0.07 |  |  |  |  |  |  |  |
| 5. Similarity | **0.03** | 0.41** | -0.23** | 0.29** |  |  |  |  |  |  |
| 6. Warmth | **0.09** | 0.52** | -0.33** | 0.26** | 0.37** |  |  |  |  |  |
| 7. Competence | **-0.14*** | 0.46** | -0.08 | 0.19** | 0.23** | 0.31** |  |  |  |  |
| 8. Masculinity | **-0.10** | 0.22** | 0.09 | 0.04 | -0.00 | 0.08 | 0.23** |  |  |  |
| 9. Secret Enjoyment | **0.09** | 0.53** | -0.23** | 0.32** | 0.31** | 0.30** | 0.35** | 0.00 |  |  |
| 10. Narcissism | **-0.07** | -0.01 | 0.42 | -0.06 | -0.09 | -0.22** | 0.03 | 0.09 | 0.07 |  |

*Note.* * *p* < .05; ** *p* < .01; + *p* < adjusted *α* = 0.006 (0.05/9). ‘Romantic Suitability’ is an index of participants’ average scores on perceived attraction, suitability for short- and long-term partnership (general and personal), and suitability for friendship (*α* = .84). *N* = 202.

## **Table S26**

*Bonferroni-corrected Spearman’s correlations for ECR-S (Avoidant) Score and Evaluations of the Selfishness Narcissist*

|  | 1 | 2 | 3 | 4 | 5 | 6 | 7 | 8 | 9 | 10 |
| --- | --- | --- | --- | --- | --- | --- | --- | --- | --- | --- |
| 1. NPI |  |  |  |  |  |  |  |  |  |  |
| 2. Romantic Suitability | **0.04** |  |  |  |  |  |  |  |  |  |
| 3. Toxic Behaviours | **0.09** | -0.22** |  |  |  |  |  |  |  |  |
| 4. Familiarity | **0.05** | 0.37** | -0.10 |  |  |  |  |  |  |  |
| 5. Similarity | **0.15*** | 0.42** | -0.41** | 0.35** |  |  |  |  |  |  |
| 6. Warmth | **0.08** | 0.46** | -0.33** | 0.26** | 0.46** |  |  |  |  |  |
| 7. Competence | **-0.05** | 0.52** | -0.37** | 0.30** | 0.39** | 0.28** |  |  |  |  |
| 8. Masculinity | **-0.13** | 0.14 | 0.05 | 0.03 | -0.10 | -0.10 | 0.12 |  |  |  |
| 9. Secret Enjoyment | **0.12** | 0.48** | -0.11 | 0.38** | 0.38** | 0.35** | 0.26** | 0.04 |  |  |
| 10. Narcissism | **-0.06** | -0.18** | 0.42** | -0.06 | -0.30** | -0.32** | -0.27** | 0.03 | -0.08 |  |

*Note.* * *p* < .05; ** *p* < .01; + *p* < adjusted *α* = 0.006 (0.05/9). ‘Romantic Suitability’ is an index of participants’ average scores on perceived attraction, suitability for short- and long-term partnership (general and personal), and suitability for friendship (*α* = .84). *N* = 202.

## **Table S27**

*Bonferroni-corrected Spearman’s correlations for ECR-S (Avoidant) Score and Evaluations of the Non-Selfishness Narcissist*

|  | 1 | 2 | 3 | 4 | 5 | 6 | 7 | 8 | 9 | 10 |
| --- | --- | --- | --- | --- | --- | --- | --- | --- | --- | --- |
| 1. NPI |  |  |  |  |  |  |  |  |  |  |
| 2. Romantic Suitability | **-0.16*** |  |  |  |  |  |  |  |  |  |
| 3. Toxic Behaviours | **0.23**+** | -0.45** |  |  |  |  |  |  |  |  |
| 4. Familiarity | **0.11** | 0.35** | -0.01 |  |  |  |  |  |  |  |
| 5. Similarity | **-0.17*** | 0.55** | -0.35** | 0.30** |  |  |  |  |  |  |
| 6. Warmth | **-0.29**+** | 0.70** | -0.40** | 0.22** | 0.53** |  |  |  |  |  |
| 7. Competence | **-0.22**+** | 0.49** | -0.28** | 0.11 | 0.44** | 0.47** |  |  |  |  |
| 8. Masculinity | **0.04** | -0.10 | 0.24** | 0.05 | -0.03 | -0.10 | 0.03 |  |  |  |
| 9. Secret Enjoyment | **-0.03** | 0.58** | -0.28** | 0.41** | 0.47** | 0.46** | 0.30** | -0.02 |  |  |
| 10. Narcissism | **-0.02** | -0.20** | 0.24** | -0.09 | -0.18* | -0.30** | -0.12 | 0.13 | -0.12 |  |

*Note.* * *p* < .05; ** *p* < .01; + *p* < adjusted *α* = 0.006 (0.05/9). ‘Romantic Suitability’ is an index of participants’ average scores on perceived attraction, suitability for short- and long-term partnership (general and personal), and suitability for friendship (*α* = .84). *N* = 202.

## **Table S28**

*Bonferroni-corrected Spearman’s correlations for ECR-S (Avoidant) Score and Evaluations of the Vanity Narcissist*

|  | 1 | 2 | 3 | 4 | 5 | 6 | 7 | 8 | 9 | 10 |
| --- | --- | --- | --- | --- | --- | --- | --- | --- | --- | --- |
| 1. NPI |  |  |  |  |  |  |  |  |  |  |
| 2. Romantic Suitability | **0.05** |  |  |  |  |  |  |  |  |  |
| 3. Toxic Behaviours | **-0.04** | -0.16* |  |  |  |  |  |  |  |  |
| 4. Familiarity | **0.19**+** | 0.49** | -0.11 |  |  |  |  |  |  |  |
| 5. Similarity | **-0.01** | 0.36** | -0.36** | 0.29** |  |  |  |  |  |  |
| 6. Warmth | **0.05** | 0.46** | -0.34** | 0.30** | 0.40** |  |  |  |  |  |
| 7. Competence | **-0.08** | 0.44** | -0.18* | 0.20** | 0.20** | 0.19** |  |  |  |  |
| 8. Masculinity | **-0.13** | -0.13 | 0.17* | -0.05 | -0.09 | -0.16* | 0.05 |  |  |  |
| 9. Secret Enjoyment | **0.20**+** | 0.53** | -0.13 | 0.44** | 0.28** | 0.26** | 0.27** | -0.09 |  |  |
| 10. Narcissism | **0.01** | -0.08 | 0.49** | -0.15* | -0.42** | -0.29** | -0.01 | 0.12 | 0.02 |  |

*Note.* * *p* < .05; ** *p* < .01; + *p* < adjusted *α* = 0.006 (0.05/9). ‘Romantic Suitability’ is an index of participants’ average scores on perceived attraction, suitability for short- and long-term partnership (general and personal), and suitability for friendship (*α* = .84). *N* = 202.

## **Table S29**

*Bonferroni-corrected Spearman’s correlations for ECR-S (Avoidant) Score and Evaluations of the Non-Vanity Narcissist*

|  | 1 | 2 | 3 | 4 | 5 | 6 | 7 | 8 | 9 | 10 |
| --- | --- | --- | --- | --- | --- | --- | --- | --- | --- | --- |
| 1. NPI |  |  |  |  |  |  |  |  |  |  |
| 2. Romantic Suitability | **-0.12** |  |  |  |  |  |  |  |  |  |
| 3. Toxic Behaviours | **0.11** | -0.22** |  |  |  |  |  |  |  |  |
| 4. Familiarity | **0.09** | 0.33** | -0.07 |  |  |  |  |  |  |  |
| 5. Similarity | **-0.08** | 0.41** | -0.23** | 0.29** |  |  |  |  |  |  |
| 6. Warmth | **0.03** | 0.52** | -0.33** | 0.26** | 0.37** |  |  |  |  |  |
| 7. Competence | **-0.05** | 0.46** | -0.08 | 0.19** | 0.23** | 0.31** |  |  |  |  |
| 8. Masculinity | **-0.23**+** | 0.22** | 0.09 | 0.04 | -0.00 | 0.08 | 0.23** |  |  |  |
| 9. Secret Enjoyment | **-0.04** | 0.53** | -0.23** | 0.32** | 0.31** | 0.30** | 0.35** | 0.00 |  |  |
| 10. Narcissism | **-0.04** | -0.01 | 0.42** | -0.06 | -0.09 | -0.22** | 0.03 | 0.09 | 0.07 |  |

*Note.* * *p* < .05; ** *p* < .01; + *p* < adjusted *α* = 0.006 (0.05/9). ‘Romantic Suitability’ is an index of participants’ average scores on perceived attraction, suitability for short- and long-term partnership (general and personal), and suitability for friendship (*α* = .84). *N* = 202.

# Supplementary Materials 8: Reporting ANOVA Results of Additional Face Comparisons (Experiment 3)

Comparing the Vain Non-Narcissist and Both Narcissists

The vain non-narcissist (vs. both narcissistic faces) was seen as warmer, more similar, and more suitable for friendship and long-term partnership (personal), as well as less narcissistic, and relationally toxic (*p*s≤.032). The vain non-narcissist was seen as more familiar than the vain narcissist (*p*=.001). However, they were also judged as less *generally* attractive, and as less *generally* suitable for short-term partnership relative to both narcissistic faces (*p*s≤.003). Furthermore, the vain non-narcissist was seen as less *personally* attractive, *personally* suitable for short-term partnership, and competent relative to the vain narcissist (*p*s≤.041). There were no differences in ratings of *general l*ong-term partnership suitability or secret enjoyment between the vain non-narcissist and both narcissistic faces, or ratings of competence, or familiarity between the vain non-narcissist and the selfish narcissist (*p*s≥.063). The vain non-narcissist was also seen as less masculine relative to the selfish narcissist (*p*<.001) but no more or less masculine than the vain narcissist (*p*=.069).

Comparing the Selfish Non-Narcissist and Both Narcissists

Finally, for comparisons between the selfish non-narcissist and both narcissistic faces, the selfish non-narcissist was seen as more attractive (general and personal), suitable for short- and long-term partnership (general and personal), and less relationally toxic relative to both narcissistic faces (*p*s≤.001). The selfish non-narcissist was also ascribed higher ratings of similarity, familiarity, warmth, competence and secret enjoyment, and lower ratings of narcissism and masculinity relative to both narcissistic faces (*p*s<.001).

Summary

In addition to being perceived as desirable relative to the selfish non-narcissist, the vain non-narcissist was also favored less relative to the narcissistic faces (particularly the vain narcissist). The vain narcissist was also seen as more competent, *personally* attractive, and *personally* desirable as a short-term partner relative to its non-narcissistic counterpart. Thus, not only does the ‘absence’ of narcissistic vanity represented in a facial image elicit lower ratings of openness, extraversion, trust, success, and political leadership, but also diminished romantic perceptions. Furthermore, once again, high rater narcissism positively predicted greater perceived similarity. This, in turn diminished negative perceptions of the faces’ toxic relationship behaviors and, in tandem with perceived familiarity, heightened judgments of romantic suitability and attraction.

# References

Gentile, B., Miller, J. D., Hoffman, B. J., Reidy, D. E., Zeichner, A., & Campbell, W. K. (2013). A test of two brief measures of grandiose narcissism: The Narcissistic Personality Inventory-13 and the Narcissistic Personality Inventory-16. *Psychological Assessment*, *25*(4), 1120–1136. https://doi.org [10.1037/a0033192](https://doi.org/10.1037/a0033192)

Haslam, C., & V. T., Montrose. (2015). Should have known better: The impact of mating experience and the desire for marriage upon attraction to the narcissistic personality. *Personality and Individual Differences, 82*, 188-192. https://doi.org/10.1016/j.paid.2015.03.032.

Jones, D. (2011). The Emotional Promiscuity Scale. In D. Fisher, C.M. Davis, W.L. Yarber,& S.L. Davis (3^rd^ Eds.). *Handbook of sexuality-related measures* (pp. 226–227). London: Routledge.

Konrath, S., Meier, B.P., & Bushman, B.J. (2014). Development and validation of the Single Item Narcissism Scale (SINS). *PLoS ONE 9*(8), e103469. <https://doi.org/10.1371/journal.pone.0103469>

Leckelt, M., Wetzel, E., Gerlach, T. M., Ackerman, R. A., Miller, J. D., Chopik, W. J., Penke, L., Geukes, K., Küfner, A., Hutteman, R., Richter, D., Renner, K. H., Allroggen, M., Brecheen, C., Campbell, W. K., Grossmann, I., & Back, M. D. (2018). Validation of the Narcissistic Admiration and Rivalry Questionnaire Short Scale (NARQ-S) in convenience and representative samples. *Psychological Assessment*, *30*(1), 86–96. https://doi.org/10.1037/pas0000433

Wei, M., Russell, D. W., Mallinckrodt, B., & Vogel, D. L. (2007). Experiences in Close Relationship Scale--Short Form (ECR, ECR-S) [Database record]. APA PsycTests. https://doi.org/10.1037/t12391-000
